# Decreased cohesin in the brain leads to defective synapse development and anxiety-related behavior

**DOI:** 10.1084/jem.20161517

**Published:** 2017-05-01

**Authors:** Yuki Fujita, Koji Masuda, Masashige Bando, Ryuichiro Nakato, Yuki Katou, Takashi Tanaka, Masahiro Nakayama, Keizo Takao, Tsuyoshi Miyakawa, Tatsunori Tanaka, Yukio Ago, Hitoshi Hashimoto, Katsuhiko Shirahige, Toshihide Yamashita

**Affiliations:** 1 Department of Molecular Neuroscience, Graduate School of Medicine, Osaka University, Osaka 565-0871, Japan; 2 Laboratory of Molecular Neuropharmacology, Graduate School of Pharmaceutical Sciences, Osaka University, Osaka 565-0871, Japan; 3 Division of Bioscience, Institute for Datability Science, Osaka University, Osaka 565-0871, Japan; 4 iPS Cell-based Research Project on Brain Neuropharmacology and Toxicology, Graduate School of Pharmaceutical Sciences, Osaka University, Osaka 565-0871, Japan; 5 Molecular Research Center for Children’s Mental Development, United Graduate School of Child Development, Osaka University, Osaka 565-0871, Japan; 6 Graduate School of Frontier Biosciences, Osaka University, Osaka 565-0871, Japan; 7 Research Center for Epigenetic Disease, Institute for Molecular and Cellular Biosciences, The University of Tokyo, Tokyo 113-0032, Japan; 8 Department of Pathology, Osaka Medical Center and Research Institute for Maternal and Child Health, Osaka 594-1101, Japan; 9 Life Science Research Center, University of Toyama, Toyama 930-0194, Japan; 10 Division of Systems Medical Science, Institute for Comprehensive Medical Science, Fujita Health University, Aichi 470-1192, Japan

## Abstract

Abnormal epigenetic regulation can cause the nervous system to develop abnormally. Here, we sought to understand the mechanism by which this occurs by investigating the protein complex cohesin, which is considered to regulate gene expression and, when defective, is associated with higher-level brain dysfunction and the developmental disorder Cornelia de Lange syndrome (CdLS). We generated conditional *Smc3*-knockout mice and observed greater dendritic complexity and larger numbers of immature synapses in the cerebral cortex of *Smc3^+/−^* mice. *Smc3^+/−^* mice also exhibited more anxiety-related behavior, which is a symptom of CdLS. Further, a gene ontology analysis after RNA-sequencing suggested the enrichment of immune processes, particularly the response to interferons, in the *Smc3^+/−^* mice. Indeed, fewer synapses formed in their cortical neurons, and this phenotype was rescued by STAT1 knockdown. Thus, low levels of cohesin expression in the developing brain lead to changes in gene expression that in turn lead to a specific and abnormal neuronal and behavioral phenotype.

## Abstract (precis)

Cohesin is associated with the developmental disorder Cornelia de Lange syndrome. Fujita et al. show that low levels of cohesin expression in the developing brain result in changes in gene expression that in turn lead to a specific and abnormal neuronal and behavioral phenotype.

## Introduction

Accumulating evidence indicates that alteration of the chromosomal architecture leads to transcriptional changes during the formation of the neuronal network (Misteli, 2007; Takizawa and Meshorer, 2008; Li et al., 2012; Kohwi and Doe, 2013; Nord et al., 2015). Abnormal epigenetic regulation of gene expression during this period causes several developmental disorders of the nervous system, such as autism spectrum disorder and Rett syndrome (Urdinguio et al., 2009; Bourgeron, 2015).

Cohesin is a highly conserved nuclear protein complex known to regulate not only higher-order chromatin organization, but also gene expression (Dorsett, 2007; Wendt et al., 2008). It is composed of four subunits (Smc1, Smc3, Scc3, and Scc1; Guacci et al., 1997; Michaelis et al., 1997; Losada et al., 1998), which form a ring structure (Nasmyth and Haering, 2005; Nasmyth, 2011). During cell division, the cohesin ring binds sister chromatids together from S phase until the subsequent anaphase, thus ensuring proper chromosomal segregation during the cell cycle.

Furthermore, cohesin is expressed not only in dividing cells but also in postmitotic cells (Wendt et al., 2008), suggesting that cohesin may also have cohesion-independent functions. In yeast, cohesin helps establish the boundaries that prevent the spread of silenced chromatin (Donze et al., 1999). In *Drosophila melanogaster*, Nipped-B, which is required to load cohesin onto DNA, mediates long-range activation of the homeobox genes *cut* and *ultrabithorax* (Rollins et al., 1999). Previous studies have indicated that cohesin functions in axon pruning of the *Drosophila* mushroom-body γ neurons. Its disruption leads to axon pruning defects in *Drosophila* (Pauli et al., 2008; Schuldiner et al., 2008), presumably through altered gene expression triggered by inhibition of cohesin, although direct evidence for this mechanism is lacking.

Cohesin also functions in the human central nervous system (CNS). Mutations that perturb its function, or those that affect the proteins that regulate it, cause Cornelia de Lange syndrome (CdLS; Krantz et al., 2004; Tonkin et al., 2004; Musio et al., 2006), a rare malformation syndrome characterized by intellectual disability, limb abnormalities, and distinctive facial features. Most of these mutations do not cause overt defects in cohesin itself or in chromosomal segregation, indicating that the disease phenotype does not result from defective cohesion. The developmental defects seen in humans might therefore be caused by altered gene expression due to the disruption of chromosomal architecture by malfunctioning cohesin; however, evidence for this hypothesis is lacking.

We postulated that epigenetic control of gene expression by cohesin is required for normal brain development and higher brain function. Here, we show that abnormally low cohesin expression in neurons caused abnormal dendrite and synapse formation in mice, which led to increased anxiety-related behavior in mice. Further, gene ontology (GO) analysis revealed that genes associated with CNS development were altered in the cortex of *Smc3^+/−^* mice. Thus, sufficient expression of cohesin must be present in the developing brain to ensure proper epigenetic control of several genes and a specific neuronal and behavioral phenotype. Cohesin thus acts as a global gene regulator and contributes to a phase of neuronal network formation.

## Results

### Generation of conditional *Smc3*-knockout mice

**Figure 1.**
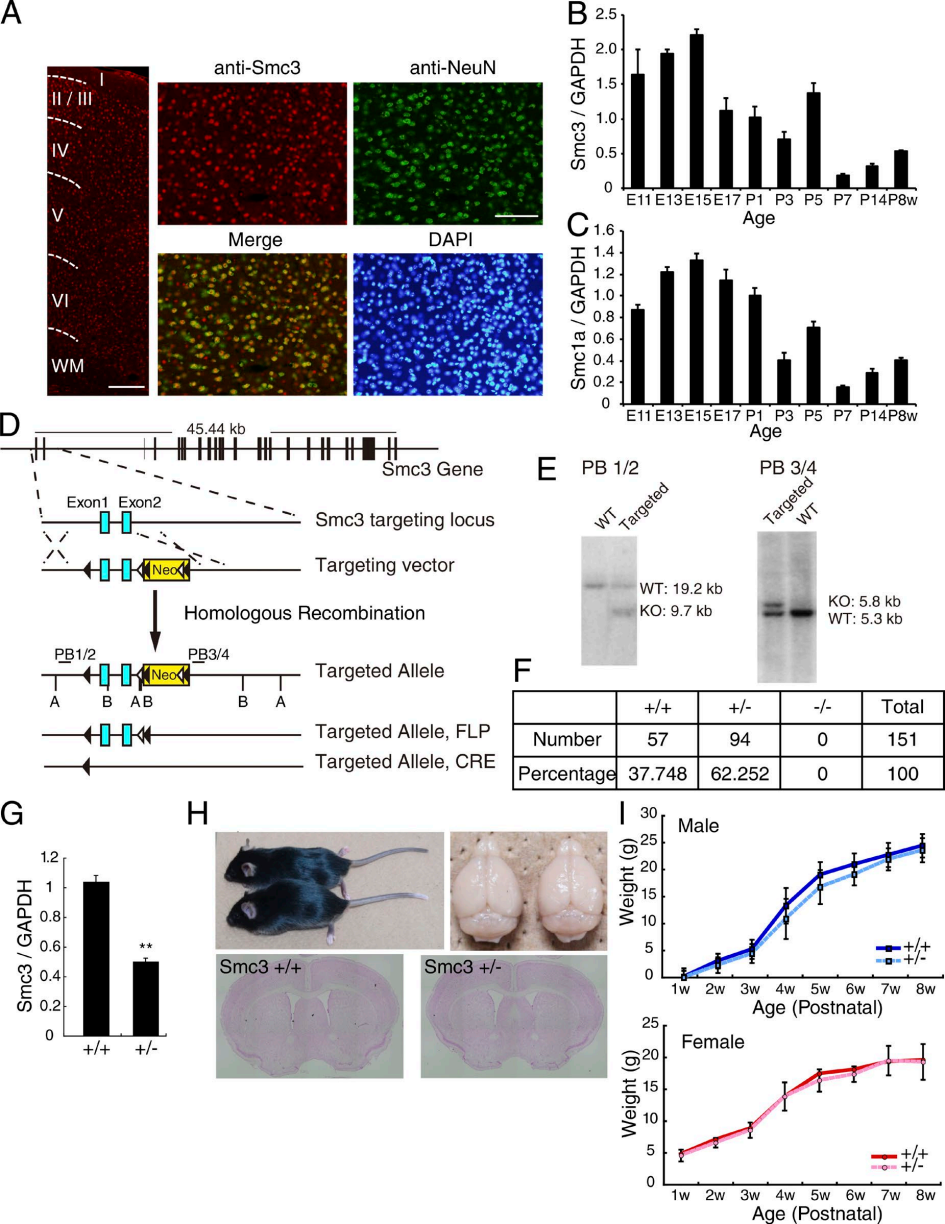
**Generation of conditional *Smc3*-knockout mice.** (A) Smc3 (red) and NeuN (green) staining in adult cerebral cortex (low-magnification image, left) and in layer II/III of the cerebral cortex (high-magnification image, right). WM, white matter. Bars: (low magnification) 50 µm; (high magnification) 100 µm. (B and C) Relative expression of Smc3 (B) and Smc1a (C) at the indicated developmental stages. *n* = 3. (D) Generation of a conditional *Smc3* knockout allele. Exons 1 and 2 were flanked by *loxP* sites (black triangles), and the neomycin resistance cassette (Neo), flanked by FRT sites (white triangles), was removed by crossing to FLPe transgenic mice. AvrII (A) and BsrGI (B) sites used for Southern blotting are indicated. Probes used for Southern blotting are depicted with black bars. (E) Representative Southern blots of genomic DNA showing targeting of the *Smc3* locus in ES cells. Genomic DNA was digested with AvrII or BsrGI and probed with PB1/2 or PB3/4. (F) Complete deletion of *Smc3* is embryonic lethal; no *Smc3*-null mice were born. (G) The levels of *Smc3* RNA expression in the brain of *Smc3^+/+^* and *Smc3^+/−^* mice. **, P < 0.01; Welch’s *t* test; *n* = 4. (H) *Smc3^+/−^* mice had normal body size and brain size. Bottom images show Nissl-stained coronal sections of brains from P6w *Smc3^+/+^* and *Smc3^+/−^* mice. (I) Body weights of *Smc3^+/+^* and *Smc3^+/−^* mice from P1w to P8w. *n* = 13, each stage. Values in graphs are expressed as the mean ± SEM.

Smc3 is the critical subunit to establish the ring structure of cohesin. We first investigated the expression of cohesin in terminally differentiated neurons by performing immunohistochemistry for Smc3 in mouse brain tissue. Brain sections from postnatal week 6 (P6w) mice were immunostained with anti-Smc3 and anti-NeuN. Smc3 was expressed ubiquitously in the brain in both NeuN-positive and NeuN-negative cells in all layers of the cerebral cortex (Fig. 1 A). The expression profiles of *Smc3* and *Smc1* transcripts in the developing brain were also examined (Fig. 1, B and C). The expression of both genes peaked at approximately embryonic day 15 (E15), when neuroblasts proliferate in the brain. Additionally, expression of both genes persisted in the brain until P8w, indicating that cohesin is expressed in post-mitotic neurons both during development and in adults.

To investigate the function of cohesin in neurons, we generated conditional *Smc3*-knockout mice. Mouse *Smc3* contains 29 exons and spans ∼45.44 kb on chromosome 19. *Smc3* was disrupted in embryonic stem (ES) cells by homologous recombination using a vector targeting exons 1 and 2 (Fig. 1 D). Targeted mutation of *Smc3* was confirmed by Southern blot hybridization and PCR of genomic DNA (Fig. 1 E). We initially deleted the gene using the CAG-Cre mouse (RBRC01828; Matsumura et al., 2004), which expresses Cre recombinase in the embryo at the zygote stage. Among the resulting offspring, mice carrying the CAG-Cre transgene with a deleted copy of the *Smc3* allele (CAG-Cre; *Smc3^+/−^*) were crossed with wild-type mice to remove the CAG-Cre transgene. The genotype of the offspring from an intercross of *Smc3^+/−^* mice did not exhibit a Mendelian ratio (Fig. 1 F). Furthermore, no *Smc3^−/−^* mice were detected at E10.5. Thus, *Smc3* deficiency causes early embryonic lethality. We then characterized the *Smc3^+/−^* mice. *Smc3* mRNA expression was reduced by 50% in the cortex of *Smc3^+/−^* mice compared with *Smc3^+/+^* mice (Fig. 1 G). *Smc3^+/−^* mice exhibited no alterations in body weight or brain size and no apparent gross abnormal phenotype in the anatomical structure of the brain (Fig. 1, H and I).

**Figure 2.**
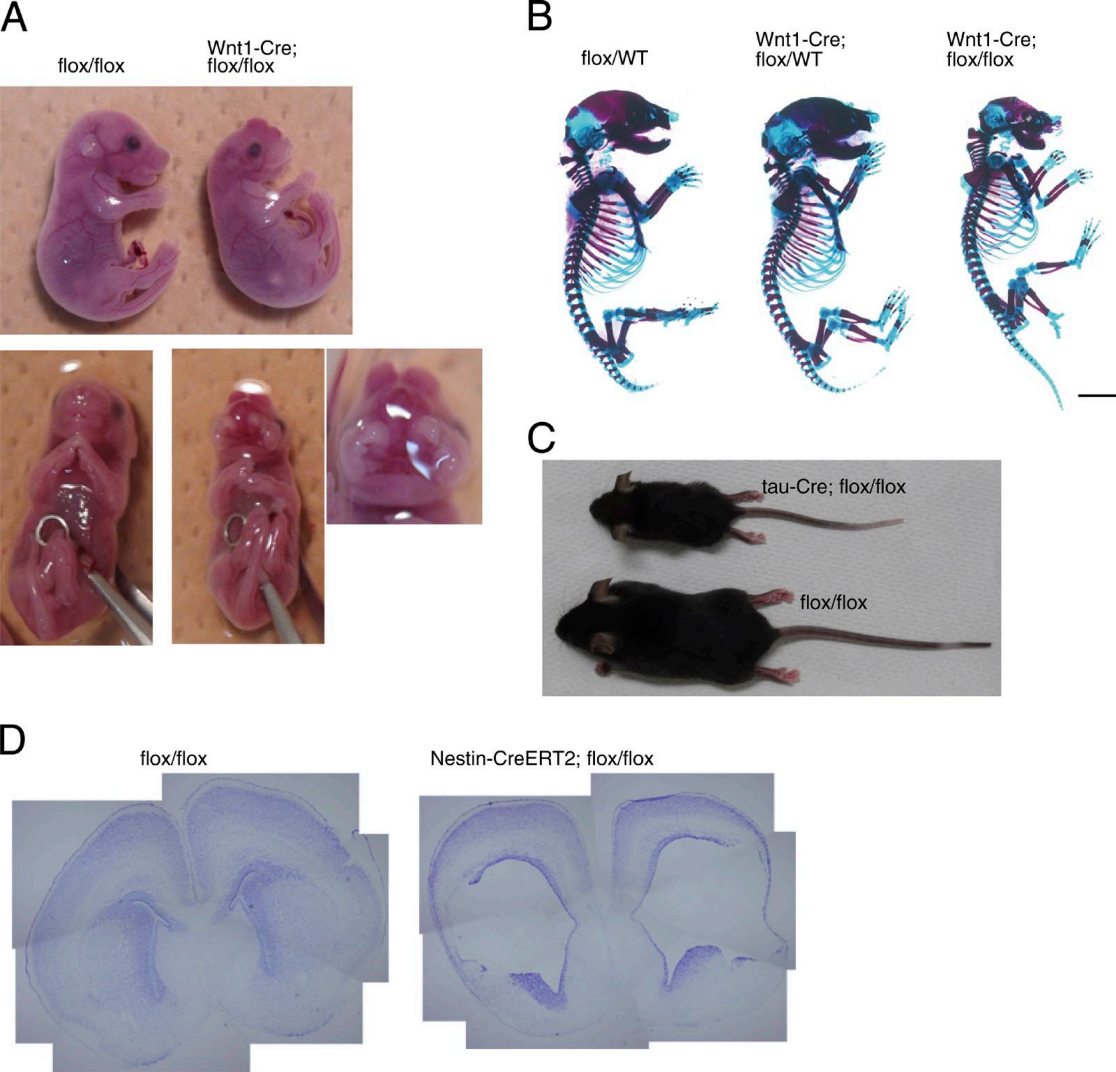
**Phenotypes of cell-specific deletion of *Smc3*.** (A) An E17.5 Wnt1-Cre; *Smc3 flox/flox* mouse showed a cleft palate (three samples). (B) Skeletal prep of a P0.5 Wnt1-Cre; *Smc3 flox/flox* mouse showed a smaller skull than that of an *Smc3 flox/flox* mouse (three samples). Bar, 2.5 mm. (C) The tau-Cre; *Smc3 flox/flox* mouse was smaller than the *Smc3 flox/flox* mouse (four samples). (D) A Nestin-Cre; *Smc3 flox/flox* mouse, in which Smc3 is deleted in neural progenitors, exhibited enlarged ventricles (six samples).

We assessed whether homozygous knockout of Smc3 in specific cell types showed a similar phenotype as CdLS. We used Wnt1-Cre mice to delete Smc3 specifically in neural crest–derived tissue and assessed craniofacial abnormalities. Wnt1-Cre; *Smc3 flox/flox* mice showed cleft palate and hypoplasia of the skull (Fig. 2, A and B). Neuron-specific deletion of Smc3 using tau-Cre mice (tau-Cre; *Smc3 flox/flox* mice) resulted in growth retardation, and these mice died by P5w (Fig. 2 C). Ablation of Smc3 in nestin-expressing neural progenitors (Nestin-CreERT2; *Smc3 flox/flox* mice) caused enlarged ventricles (Fig. 2 D). Collectively, the phenotypes observed in these mice appear to be associated with the clinical signs of CdLS.

### Cohesin regulates dendritic development in the cerebral cortex

**Figure 3.**
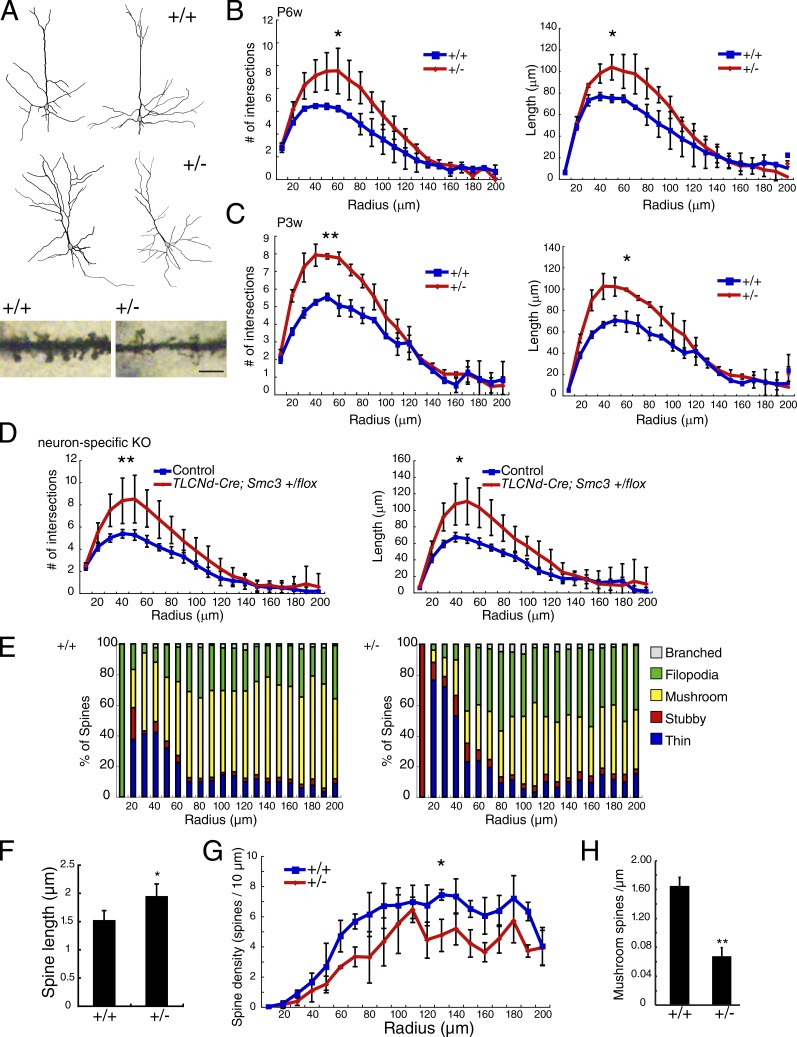
**Abnormal dendrite development in the *Smc3^+/−^* mouse cerebral cortex.** (A, top and middle) Neurolucida drawings of typical dendritic arbors of cortical layer II/III pyramidal neurons from Golgi-impregnated brain sections of *Smc3^+/+^* and *Smc3^+/−^* mice. (Bottom) Representative images of Golgi-stained dendrites. Bar, 5 µm. (B and C) Sholl analysis revealed greater neuronal complexity and dendrite length in *Smc3^+/−^* mice than in *Smc3^+/+^* mice. Brain sections were prepared from P6w (B) or P3w (C) mice. *, P < 0.05; **, P < 0.01; two-way ANOVA; *n* = 4. (D) Sholl analysis of dendrites after neuron-specific deletion of *Smc3* in heterozygous knockout (TLCN-Cre; *Smc3^+^/flox* mice) mice, P6w. *, P < 0.05; **, P < 0.01; two-way ANOVA; *n* = 6. (E) Classification of spines on apical dendrites of cortical layer II/III pyramidal neurons as branched, filopodial, mushroom, stubby, or thin. *n* = 3. (F) Spine length of cortical layer II/III pyramidal neurons is longer in *Smc3^+/−^* mice. *, P < 0.05; Welch’s *t* test; *n* = 4. (G) Sholl analysis revealed lower mushroom-type spine density in *Smc3^+/−^* mice at P6w. *, P < 0.05; two-way ANOVA; *n* = 4. (H) Mushroom-type spine density in layer II/III pyramidal neurons from *Smc3^+/−^* mice was lower than that of *Smc3^+/+^* pyramidal neurons. **, P < 0.01; Welch’s *t* test; *n* = 4. Values in graphs are expressed as the mean ± SEM.

We consider haplodeficient mice, which exhibit higher brain dysfunction, as a model of CdLS. Because abnormal synapse formation is observed in cortical layer II/III of some animal models of higher brain dysfunction (Hayashi et al., 2004; Cruz-Martín et al., 2010), we focused our analysis on individual pyramidal neurons in these layers. To investigate the effects of abnormally low *Smc3* expression on neuronal morphology, we performed Golgi staining of brain tissue obtained from *Smc3^+/+^* and *Smc3^+/−^* mice at P6w. Sholl analysis (Sholl, 1953) revealed that dendritic arbor complexity was greater in *Smc3^+/−^* mice than in *Smc3^+/+^* mice (Fig. 3, A–C).

To identify whether the *Smc3^+/−^* phenotype resulted from abnormal neuronal Smc3, we generated neuron-specific heterozygous *Smc3*-knockout mice (TLCN-Cre; *Smc3^+^/flox* mice) and again observed greater dendritic arbor complexity (Fig. 3 D). This indicated that a sufficient level of neuronal Smc3 is required for proper dendritic development.

We then analyzed the structure of apical dendritic spines in cortical layer II/III at P8w. The percentage of mushroom-type mature spines was lower in the apical dendrites of *Smc3^+/−^* mice than in *Smc3^+/+^* mice (Fig. 3 E). Additionally, the percent of thin- and filopodia-type immature spines was higher in the apical dendrites of *Smc3^+/−^* mice. Reflecting this structural alteration, spines were longer in *Smc3^+/−^* mice (Fig. 3 F). The percentage of stubby spines did not differ between *Smc3^+/+^* and *Smc3^+/−^* mice. Additionally, the density of mushroom-type mature spines was significantly lower in *Smc3^+/−^* mice than in *Smc3^+/+^* mice (Fig. 3, G and H). Thus, abnormally low expression of neuronal Smc3 led to anatomically perturbed synapse maturation in cortical layer II/III.

### Reduced cohesin expression leads to impaired synapse maturation

**Figure 4.**
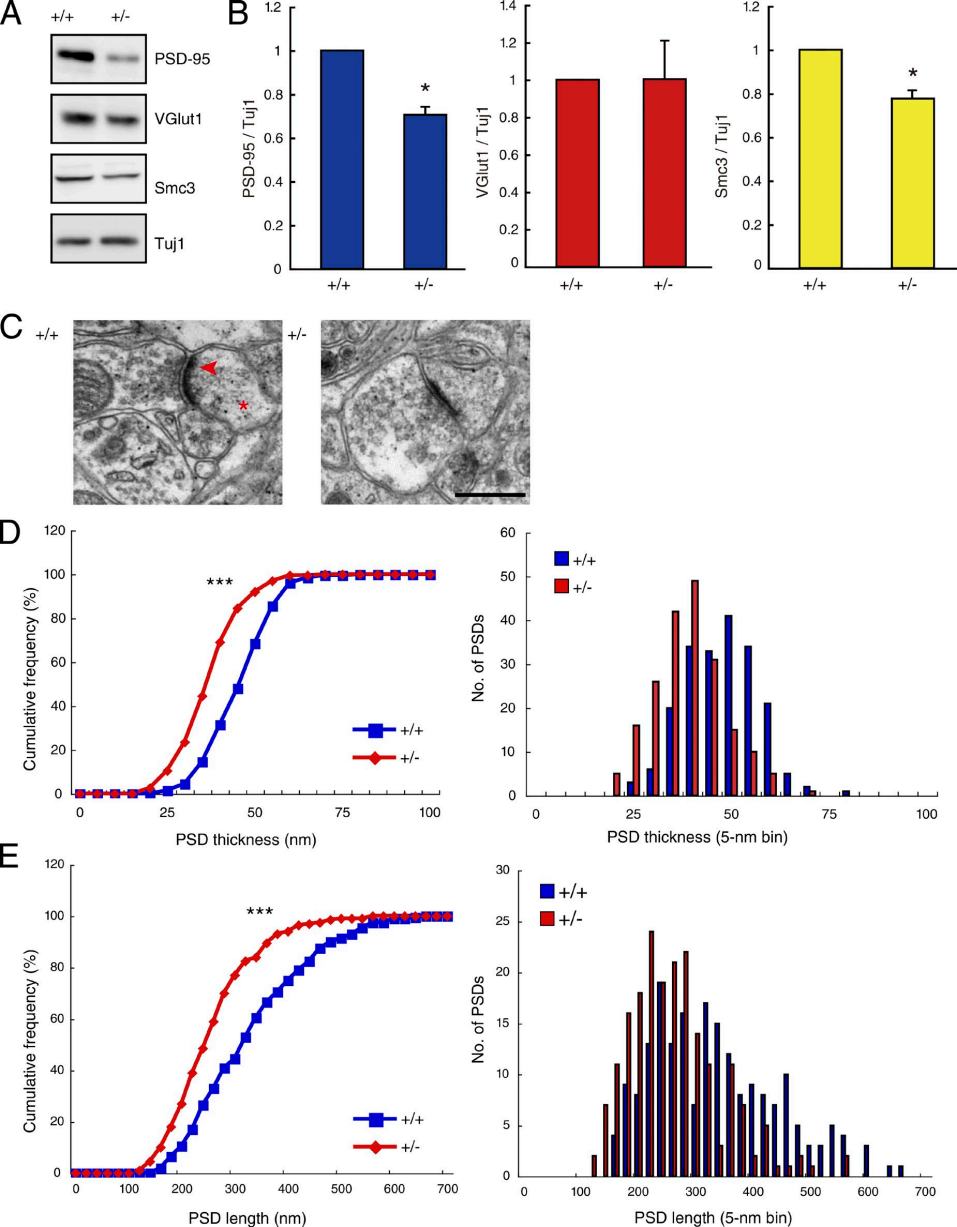
**Impaired synaptic maturation in *Smc3^+/−^* mice.** (A and B) Western blots (A) and relative expression levels (B) of synaptic markers and Smc3. Expression of PSD-95 was lower in the cortex of *Smc3^+/−^* mice. *, P < 0.05; Welch’s *t* test; *n* = 4. (C) Electron micrographs of synapses from the cortices of *Smc3^+/+^* and *Smc3^+/−^* mice. The images demonstrate synaptic contacts featuring presynaptic vesicles, postsynaptic densities (arrowhead), and dendritic spines (asterisk). Bar, 500 nm. (D and E) Reduced thickness (D) and length (E) of postsynaptic densities in *Smc3^+/−^* mice. ***, P < 0.0001; Kolmogorov–Smirnov test; *n* = 200 postsynaptic densities each from four *Smc3^+/+^* and four *Smc3^+/−^* mice. Values in graphs are expressed as the mean ± SEM.

The aforementioned morphological results prompted us to investigate the effect of cohesin on synapse formation. We observed a lower expression of the excitatory postsynaptic protein postsynaptic density 95 (PSD-95) in *Smc3^+/−^* mice than in *Smc3^+/+^* mice but did not detect any significant change in the level of the excitatory presynaptic protein VGlut1 (Fig. 4, A and B). Furthermore, electron micrographic analysis revealed significantly thinner and shorter postsynaptic densities in *Smc3^+/−^* mice at P6w (Fig. 4, C–E). Thus, if the required level of cohesin is absent, proper development of synapses in the cerebral cortex cannot occur. These observations suggest that cohesin expression in terminally differentiated neurons must be strictly controlled for the formation of a precise neuronal network in the cerebral cortex during development.

### *Smc3^+/−^* mice exhibit excessive anxiety-related behavior

Heterozygous mutations in cohesin or cohesin regulators are linked to CdLS, which is characterized by stunted growth, intellectual disability, and heightened anxiety. We therefore performed a battery of behavioral tests (Imayoshi et al., 2008) on *Smc3^+/−^* mice using adult male mice at least 11 wk old.

**Figure 5.**
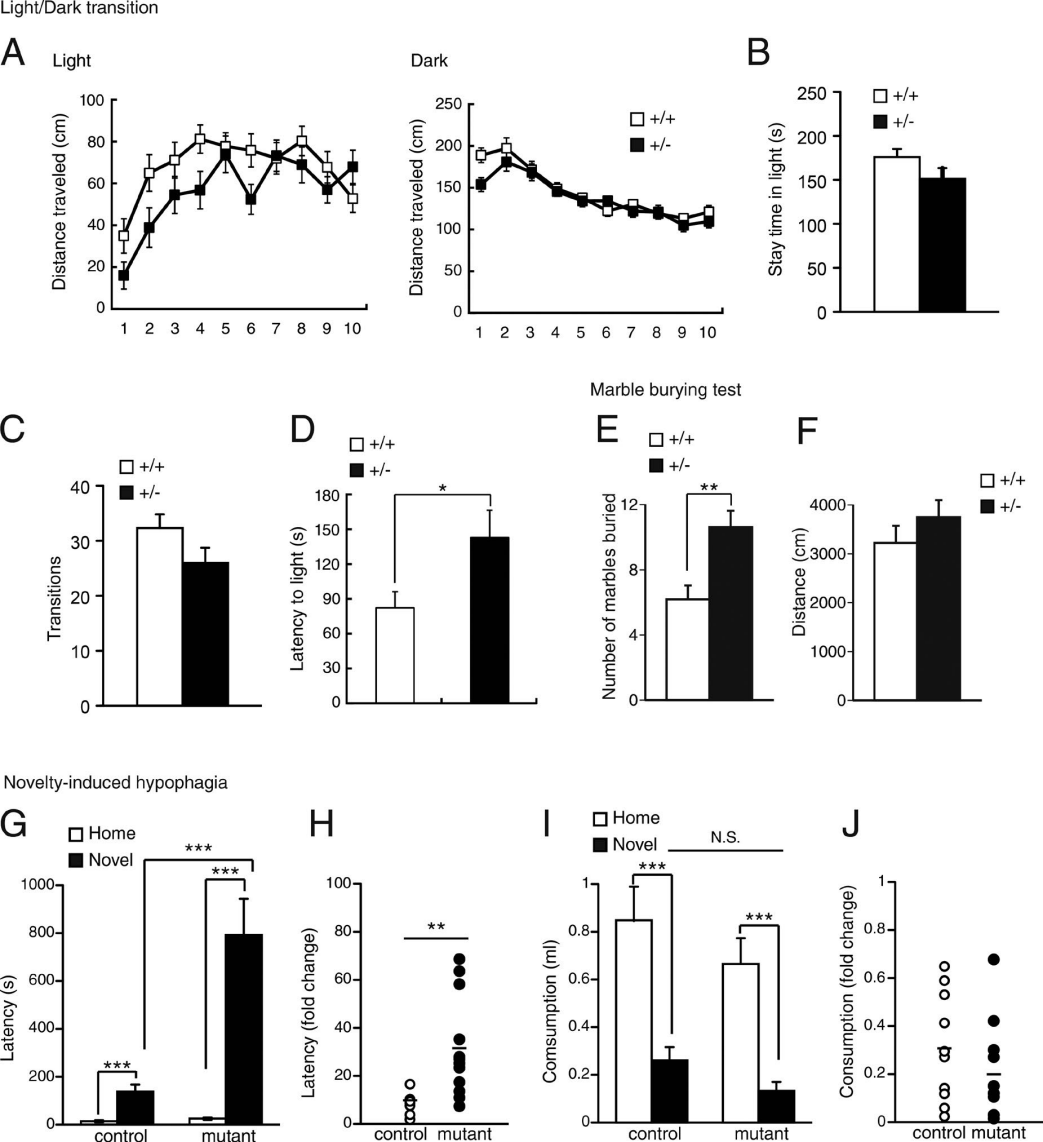
**Heightened anxiety-related behavior in *Smc3^+/−^* mice.** (A–D) Results of the light/dark transition test. The distances traveled in the light or dark boxes (A), the stay time in the light box (B), and the number of transitions (C) were not different between *Smc3^+/+^* and *Smc3^+/−^* mice. *Smc3^+/−^* mice displayed longer latency to first entry into the light side (D). *, P < 0.05, unpaired Student’s *t* test; *n* = 23 mice per genotype. (E and F) The number of marbles buried (E) and the total distance traveled (F) during the marble-burying test. *Smc3^+/−^* mice buried more marbles (E); however, we observed no significant difference between mouse groups regarding the total distance (F). **, P < 0.01; unpaired Student’s *t* test; *n* = 19 mice per genotype. (G–J) Results of the novelty-induced hypophagia test. The latency to begin drinking in a novel cage was greater in *Smc3^+/−^* mice (G and H). Consumption in the novel cage was lower (I), but no significant difference was observed in the fold change of consumption (J). **, P < 0.01; ***, P < 0.001; unpaired Student’s *t* test; *n* = 11–12 mice per group; N.S., not significant. Values in graphs are expressed as the mean ± SEM.

In the light/dark transition test (Takao and Miyakawa, 2006), whereas the distance traveled, time spent in the light box, and number of transitions did not differ between *Smc3^+/+^* and *Smc3^+/−^* mice (Fig. 5, A–C), the latency of *Smc3^+/−^* mice to cross into the light box was significantly greater (Fig. 5 D), which suggests enhanced anxiety in *Smc3^+/−^* mice. We therefore further analyzed anxiety-like behavior. The marble-burying test is correlated with anxiety-like and repetitive behavior (Njung’e and Handley, 1991; Thomas et al., 2009). Compared with *Smc3^+/+^* mice, *Smc3^+/−^* mice exhibited more marble-burying behavior, although the total distance traveled was similar between groups (Fig. 5, E and F). Novelty-induced hypophagia has also been used to measure anxiety-like behavior (Dulawa and Hen, 2005). In this test, mice are trained to approach a reward (sweetened condensed milk) in their home cage and are then placed in a novel, brightly lit cage. The latency to drinking and the volume of milk consumed are used as measures of anxiety-related behavior. *Smc3^+/−^* mice exhibited a significantly greater latency to drink in the novel cage compared with *Smc3^+/+^* mice (Fig. 5, G–J). Thus, three separate tests indicate that, as in patients with CdLS (Basile et al., 2007; Richards et al., 2009; Nakanishi et al., 2012), low expression of cohesin leads to heightened anxiety-related behavior. We detected no significant differences in locomotion, nociception, social behavior, motor coordination, depression-like behavior, or spatial memory (Figs. S1, S2, and S3). Therefore, insufficient expression of cohesin induces a surprisingly specific behavioral phenotype, suggesting that it is required for the development of a neuronal network involved in specific higher brain function.

**Figure 6.**
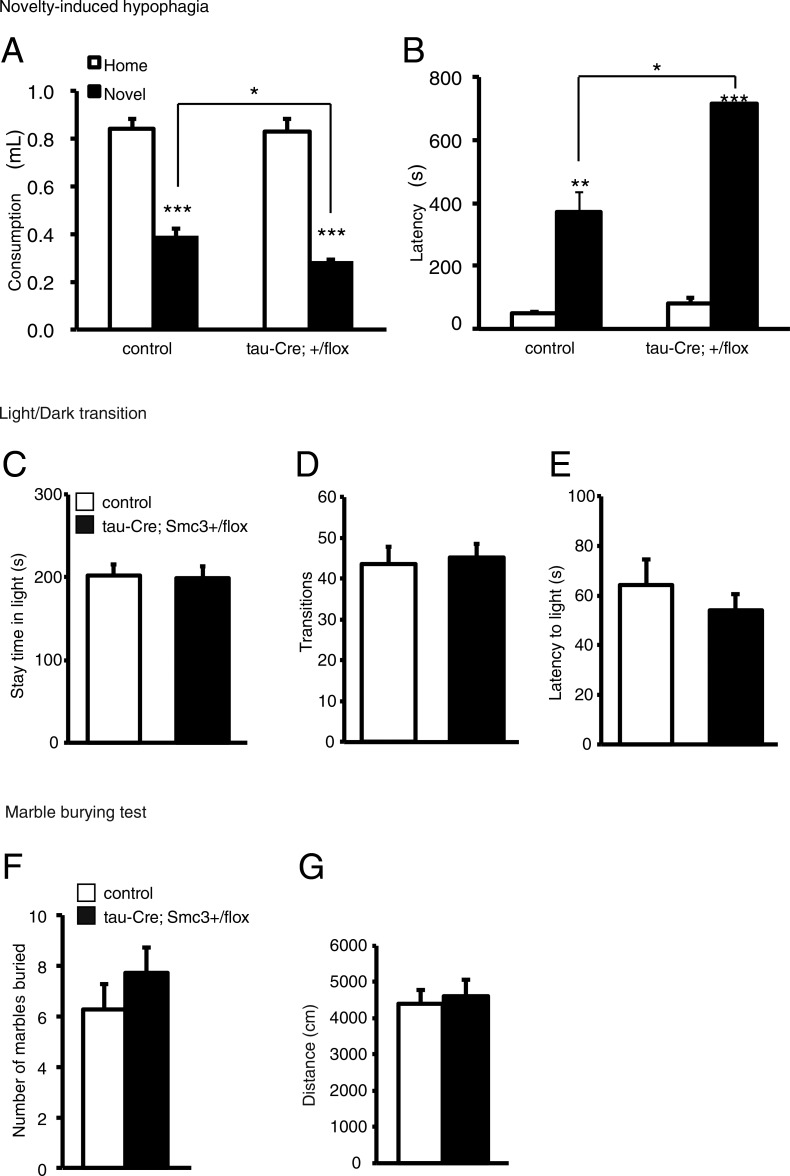
**Increased anxiety-related behavior in neuron-specific *Smc3* deletion mice.** (A and B) Anxiety-related behaviors were assessed in neuron-specific heterozygous Smc3 knockout mice. tau-Cre; *Smc3^+^/flox* mice exhibited increased anxiety-like behavior in novelty-induced hypophagia test. (C–G) Light/dark transition and marble-burying tests did not detect the significant differences between *Smc3 flox/flox* mice and tau-Cre; *Smc3^+^/flox* mice. *, P < 0.05; **, P < 0.01; ***, P < 0.001; ANOVA followed by Bonferroni–Dunn test (A and B), unpaired Student’s *t* test (C–G); *n* = 15 (*Smc3 flox/flox* mice), 13 (tau-Cre; *Smc3^+^/flox* mice). Values in graphs are expressed as the mean ± SEM.

We further investigated whether decreased expression of neuronal Smc3 produced anxiety-like behavior. Neuron-specific heterozygous *Smc3*-knockout mice (tau-Cre; *Smc3^+^/flox* mice) also showed increased anxiety-like behavior in a novelty-induced hypophagia test (Fig. 6, A and B). However, the light/dark transition test or the marble-burying test did not detect the difference between control and tau-Cre; *Smc3^+^/flox* mice (Fig. 6, C–G). These results suggest that loss of cohesin function in neurons increases anxiety-like behavior, particularly in the novelty environment.

### CdLS induces morphological changes in brain tissue

**Figure 7.**
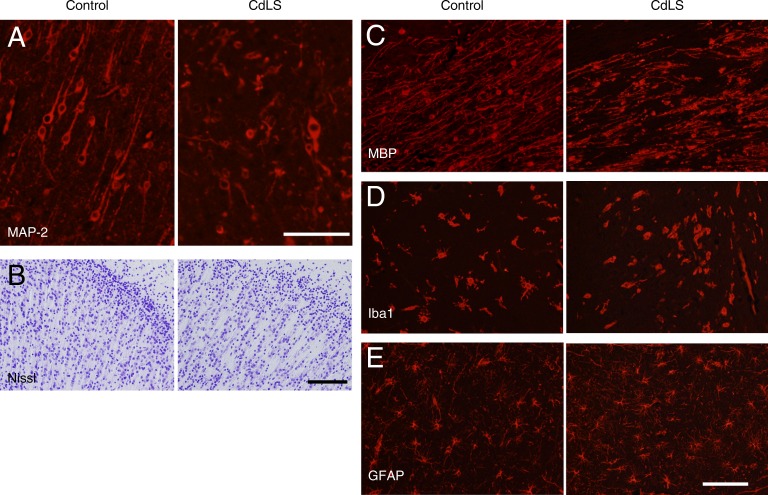
**Morphological abnormalities in autopsied brain tissue from stillborns with CdLS.** (A) Immunostaining for MAP-2 in the cortex of CdLS and control brains. (B) Nissl staining in cortical layer II/III of CdLS and age-matched control brains. (C–E) Immunostaining for MBP (C), Iba1 (D), and GFAP (E) in the cortex of CdLS and control brains. Number of samples was two. Bars: (A and C–E) 100 µm; (B) 200 µm.

The anxiety-related findings suggest an analogy between the behavioral phenotype of mice with abnormally low cohesin expression and individuals with CdLS. However, the pathological features of the CdLS brain have not been thoroughly investigated. Thus, we next analyzed autopsied cortical brain tissue from CdLS that were stillborn at 39 wk of pregnancy by performing immunohistochemical analysis of dendritic arborization. Microtubule-associated protein 2 (MAP-2) staining revealed that the dendritic arbors in the CdLS brain were thin, tortuous, and fragmented (Fig. 7 A), consistent with our observations of dendritic arbors in *Smc3^+/−^* mice (Fig. 3 A). With Nissl staining, the density of neurons was lower in the brains of individuals with CdLS than in control stillborn individuals free of neurological disease (Fig. 7 B). We also performed immunohistochemistry for myelin basic protein (MBP), ionized calcium-binding adapter molecule 1 (Iba1), and glial fibrillary acidic protein (GFAP) to examine oligodendrocytes, microglia, and astrocytes, respectively. The density of processes on MBP-positive oligodendrocytes was lower in CdLS brains (Fig. 7 C), suggesting defective myelin formation. Iba1-positive cells were slightly rounder in CdLS brains, which suggested that microglia might have been activated (Fig. 7 D). Additionally, the number of GFAP-positive astrocytes was higher in CdLS brains. Intriguingly, most of the GFAP-positive cells in the CdLS brains had a greater number of processes and thus appeared to be activated (Fig. 7 E). These results suggest that attenuated cohesin function in the CdLS brain is associated with defective neuronal network formation with attenuated myelin formation and activation of microglia and astrocytes.

### Cohesin regulates gene expression during CNS development

**Figure 8.**
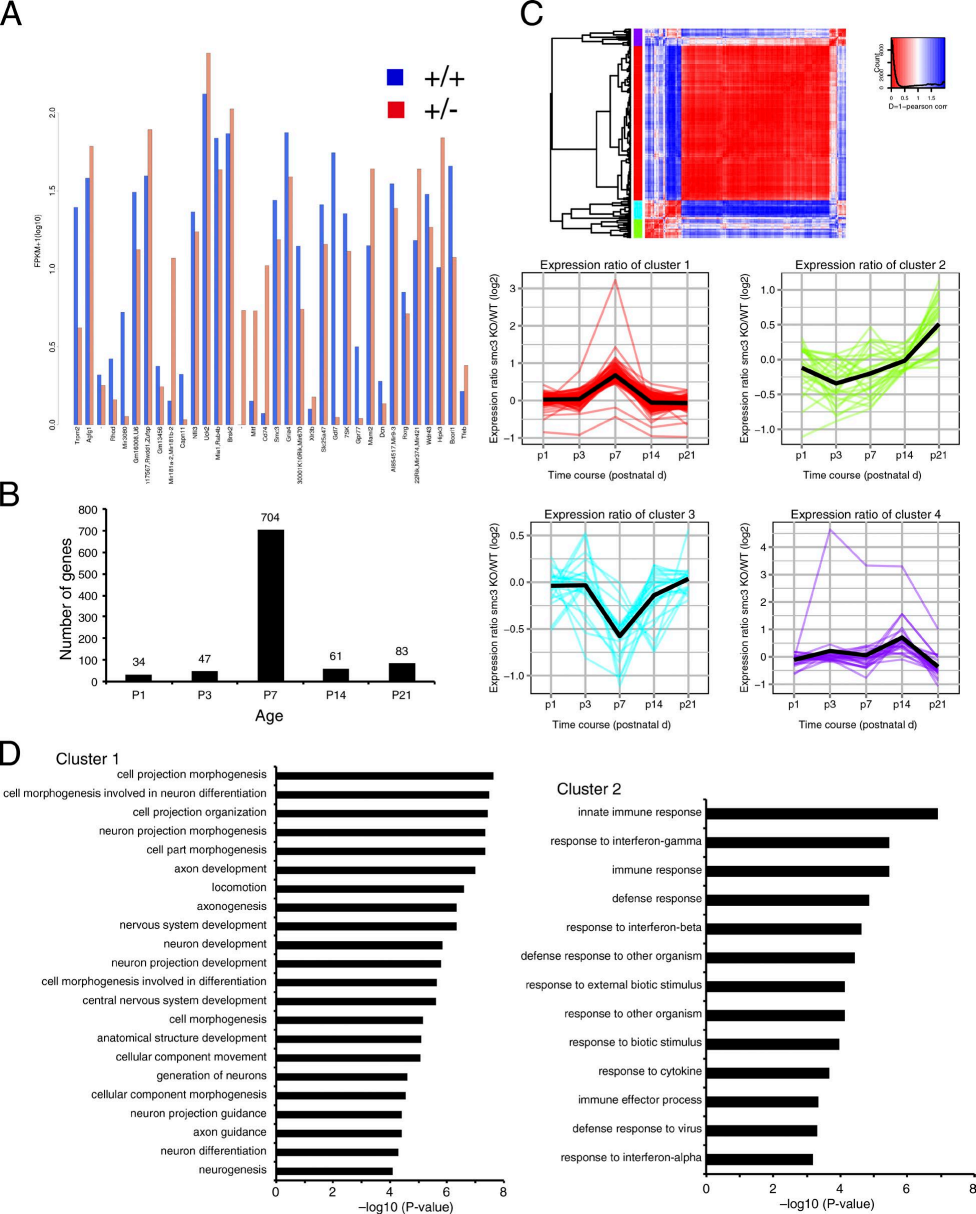
**Transcriptional landscape of *Smc3^+/−^* mice.** (A) Expression levels of genes that were significantly different between *Smc3^+/−^* and *Smc3^+/+^* mice. RNA was prepared from the P1 cortices of *Smc3^+/+^* or *Smc3^+/−^* mice, and gene expression levels were analyzed by RNA-sequencing. Fragments per transcript kilobase per million fragments mapped (FPKM) values were analyzed with three replicates. (B) Time-course analysis of the number of genes that were differentially expressed in *Smc3^+/−^* compared with *Smc3^+/+^* mice. (C) Hierarchical clustering of 290 isoforms that were altered (q-value <0.05) in the cortices of *Smc3^+/−^* mice at P1, P3, P7, P14, and P21. (D) GO terms significantly enriched in clusters 1 and 2. The x axis shows −log_10_ (p-value). *n* = 4 (P1); *n* = 3 (P3, P7, and P14).

Because regulation of gene expression is considered to be an important function of cohesin in terminally differentiated cells (Wendt et al., 2008), we suspected that the phenotype associated with the neuron-specific *Smc3*-knockout mouse depends on cohesin-mediated gene expression during development. To test this hypothesis, we performed a gene expression analysis to identify the genes responsible for the phenotype, beginning with RNA-sequencing of cortical tissue samples from postnatal day 1 (P1) *Smc3^+/+^* or *Smc3^+/−^* mice (Fig. 8 A and Table S1). We then focused on the time course of expression during dendritic formation (P1–P21). We found that in the cortex of *Smc3^+/−^* mice, many more genes were significantly altered relative to *Smc3^+/+^* mice at P7 when synapse formation was dynamically occurred in the brain than at P1, P3, P14, or P21 (Fig. 8 B). Therefore, cohesin acts as a global gene regulator at a specific time point during development, which may explain why it plays such a specific role in cortical network formation.

To analyze the genes that exhibited altered expression during development, we performed clustering analysis in which 290 genes were hierarchically grouped and subsequently classified into four clusters (Fig. 8 C). To identify the characteristics of each cluster, we performed GO analysis. GO terms were significantly enriched in clusters 1, 2, and 4. The p-values of the GO enrichments in clusters 1 (P < 8.23e-05) and 2 (P < 6.76e-04) are shown in Fig. 8 D. The GO analysis for cluster 1 indicated enrichment for neuronal development, which might thus be responsible for the phenotype of *Smc3^+/−^* mice.

**Figure 9.**
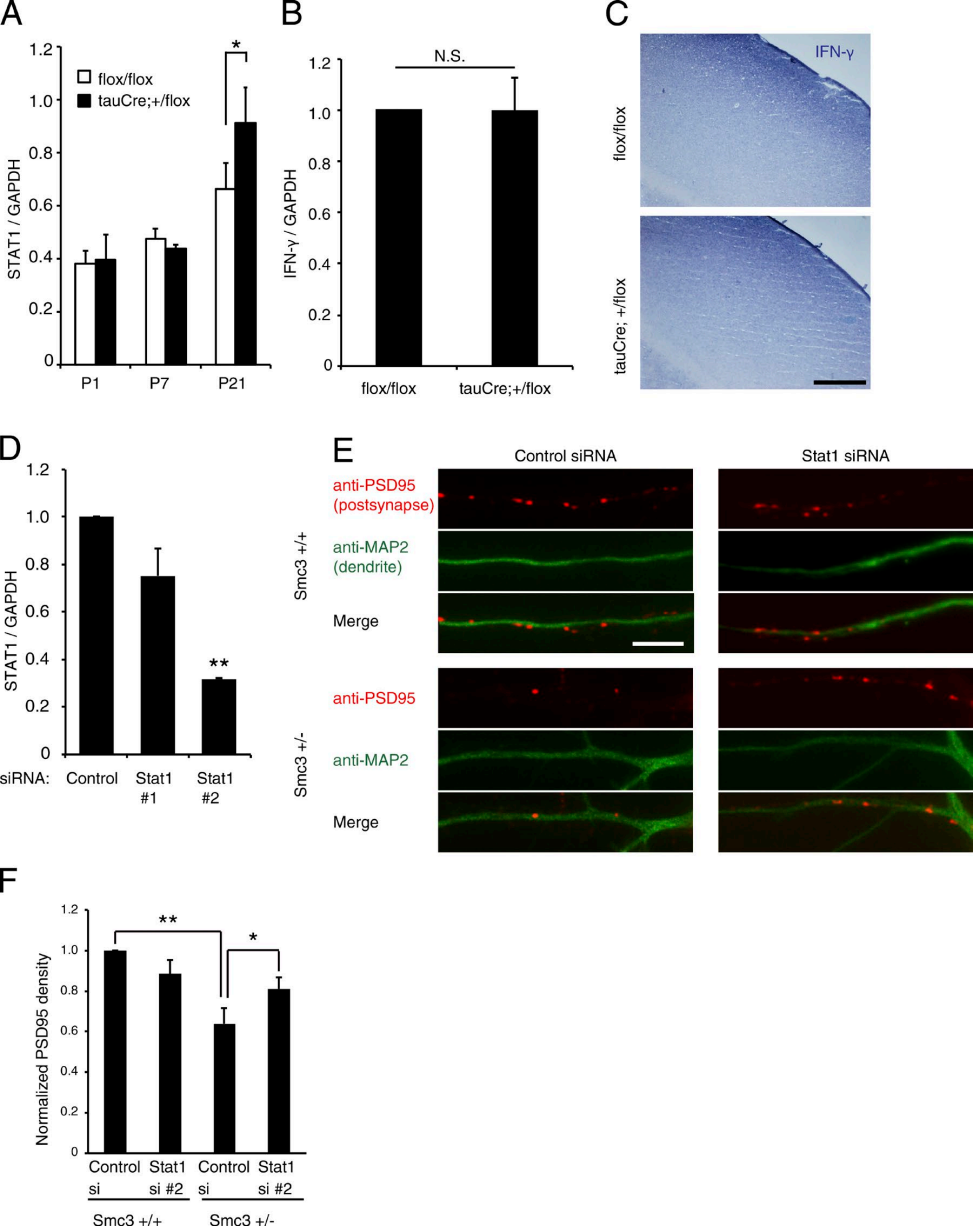
**Knockdown of Stat1 attenuates the reduction of synapse formation in *Smc3^+/−^* mice.** (A) Stat1 expression in the cortex of *Smc3 flox/flox* and tau-Cre; *Smc3^+^/flox* mice. *, P < 0.05; one-way ANOVA, followed by Scheffe’s multiple comparison test; *n* = 3. (B) IFN-γ expression in cultured astrocytes prepared from *Smc3 flox/flox* or tau-Cre; *Smc3^+^/flox* mice. N.S., not significant; *n* = 3. (C) In situ hybridization of IFN-γ mRNA in sagittal sections of P21 mouse brains. No obvious differences in IFN-γ expression between the cortices of *Smc3 flox/flox* and tau-Cre; *Smc3^+^/flox* mice were observed. (D) siRNA-mediated knockdown of STAT1 mRNA. Cortical neurons were transfected with the indicated siRNA. Total RNA isolated 72 h after transfection was analyzed by real-time PCR. **, P < 0.01; one-way ANOVA, followed by Scheffe’s multiple comparison test; *n* = 3. (E and F) Inhibition of STAT1 expression partially rescued impaired synaptic formation in *Smc3^+/−^* mice. Cortical neurons prepared from *Smc3^+/+^* or *Smc3^+/−^* mice were transfected with control or STAT1 siRNA and were cultured for 21 d. (E) Representative images showing the distribution of PSD-95 signals (red, postsynaptic marker). Dendrites (green) are extended proximal to distal, right to left. Bars: (C) 200 µm; (E) 10 µm. (F) Lower PSD-95 density in *Smc3^+/−^* mice was prevented by STAT1 knockdown. *, P < 0.05; **, P < 0.01; one-way ANOVA, followed by Scheffe’s multiple comparison test; *n* = 3. Values in graphs are expressed as the mean ± SEM.

The GO analysis for cluster 2 indicated enrichment for immune processes, particularly for the response to interferons. Interferon signaling has been implicated in the regulation of dendritic and synaptic formation through STAT1 (Kim et al., 2002). Furthermore, we found that *Stat1* expression was higher in the cortex of *Smc3^+/−^* mice at P21 (Table S2, cluster 2). *Stat1* expression was also increased in the cortex of neuron-specific heterozygous *Smc3*-knockout (tau-Cre; *Smc3^+^/flox*) mice compared with *Smc3 flox/flox* mice (Fig. 9 A). However, the expression level of IFN-γ, which activates Stat1, was not different between *Smc3 flox/flox* and tau-Cre; *Smc3^+^/flox* mice (Fig. 9, B and C), suggesting that cohesin function seems to be required for the intracellular responses in neurons, but not for ligand expression.

These findings prompted us to examine whether knockdown of *Stat1* could rescue the abnormal phenotype in *Smc3^+/−^* mice. To assess this, we looked at the number of cortical synapses in *Smc3^+/−^* mice. Transfection of *Stat1* siRNA into cortical neurons led to lower STAT1 expression (Fig. 9 D). Cortical neurons were cultured for 21 d and then immunostained with anti–PSD-95 and anti-MAP2 antibodies. Numbers of PSD-95–positive puncta in cortical neurons were less in *Smc3^+/−^* mice than in wild-type mice, and this phenotype was partially rescued by *Stat1* siRNA transfection (Fig. 9, E and F). These results suggest that cohesin regulates gene expression including STAT1 that eventually contributes to the maturation of synapses. As *Stat1* knockdown only partially rescued phenotype, other molecules (e.g., the genes in clusters 1 and 2; Table S2) likely contribute to the *Smc3^+/−^* phenotype.

**Figure 10.**
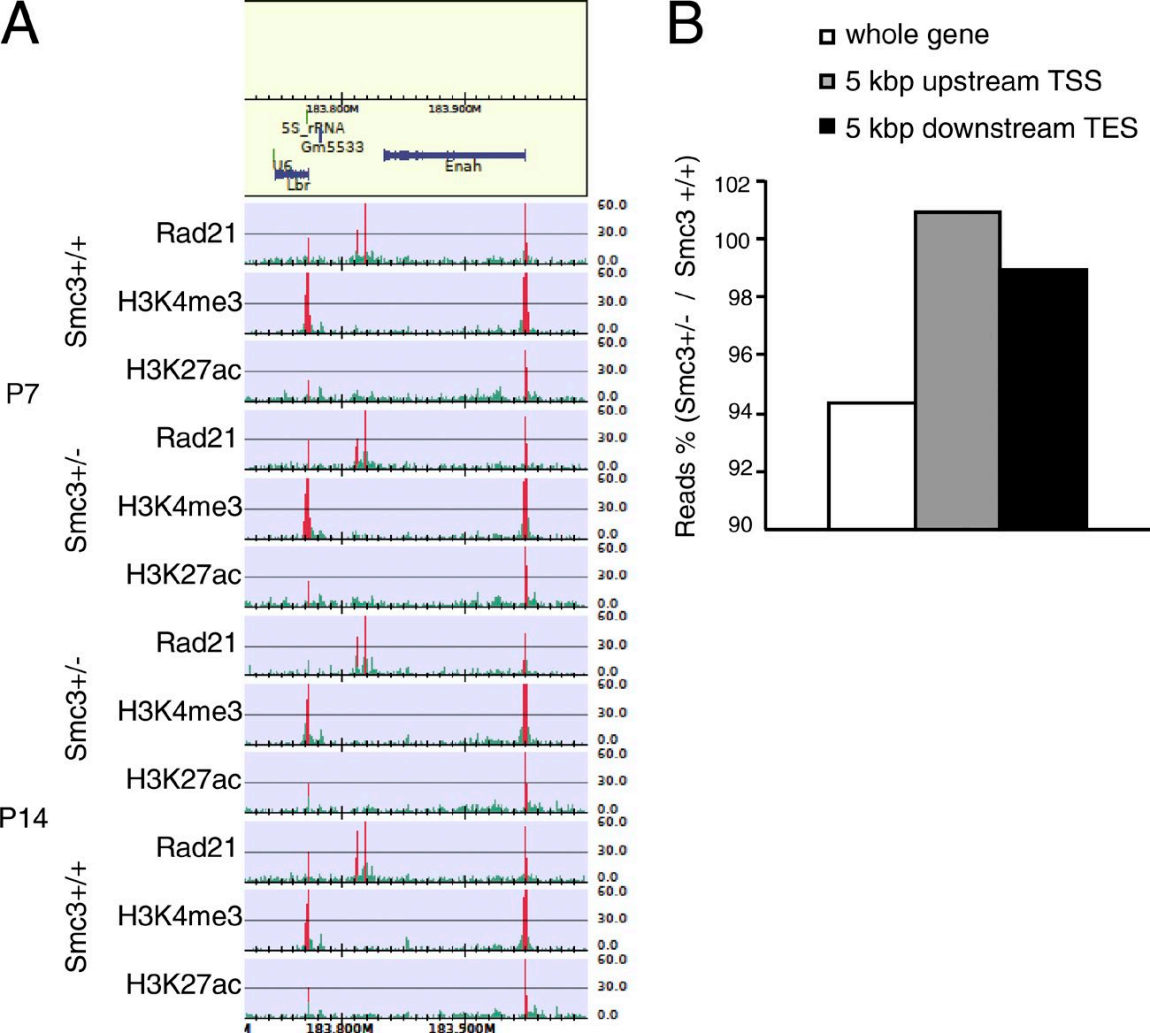
**Cohesin occupancy inside DEGs tends to be decreased.** (A) Representative data of ChIP-seq. Regions in which signals were significantly enriched are in red. Binding profiles for H3K4me3, H3K27ac, and Rad21 in the cortices of *Smc3^+/+^* and *Smc3^+/−^* mice are shown. (B) Rad21 distribution changes around DEGs at P14. Relative changes in Rad21 read between *Smc3^+/+^* and *Smc3^+/−^* mice were lower inside DEGs in cluster 2. Number of samples was two (*Smc3^+/+^*, *Smc3^+/+^* each). TSS, transcription start site; TES, transcription end site.

We further examined genome-wide histone modifications and the distribution of cohesin by chromatin immunoprecipitation (IP [ChIP]) with high-throughput sequencing (ChIP-seq). H3K4me3 is related to active promoters, and H3K27ac is related to both active promoters and enhancers (Shlyueva et al., 2014). We could not detect significant changes in the status of histone markers or the occupancy of the cohesin complex protein, Rad21 (Fig. 10 A). To enhance the positional resolution of ChIP-seq, we used spike-in samples. Rad21 distribution was altered inside but not outside differentially expressed genes (DEGs) of *Smc3^+/−^* mice at P14 (Fig. 10 B). Thus, lower cohesin occupancy inside the gene may alter gene expression during development, thus leading to impaired formation of dendrites and synapses.

## Discussion

We have demonstrated that the haplodeficiency of cohesin leads to defective dendrite development and synapse maturation in the cerebral cortex of mice. Moreover, mice with reduced cohesin expression exhibit heightened anxiety-related behavior. This phenotype is consistent with the part of symptoms of individuals with CdLS, who frequently exhibit intellectual disability, increased anxiety, and seizures. Furthermore, individuals with CdLS are frequently hyperactive (Berney et al., 1999), which was also observed in heterozygous *Smc3*-knockout mice during the dark part of the diurnal cycle in the home cage (Fig. S3, q and r). However, we could not detect the phenotype reflecting intellectual disability. To assess this point, we need to perform various behavior tests for learning and memory abilities. Because increased anxiety-like behavior was also detected in neuron-specific Smc3 heterozygous deficient mice (Fig. 6), the phenotype induced by decreased Smc3 may be caused by a cell-autonomous mechanism. Some anxiety-related behavior tests suggest increased anxiety in *Smc3^+/−^* mice, whereas other tests, such as the elevated plus maze, did not indicate increased anxiety. These findings suggest that the specific neuronal network seems to be affected by Smc3 deficiency. Altered dendritic and synapse formation was also observed in the prefrontal cortex, which regulates higher-order brain function, suggesting that the phenotype of *Smc3^+/−^* mice might be associated with disorganization in the prefrontal area.

In the current study, neuron-specific *Smc3* knockout led to enhanced dendritic complexity. Analysis revealed that the abnormal neuronal and behavioral phenotype was related to altered control of gene expression that resulted from insufficient levels of cohesion. This result provides clear evidence of a critical role for cohesin in postmitotic neurons and confirms the existence of a chromatid-cohesin–independent function of cohesin in CNS development. Reduced cohesin in the brain leads to defective epigenetic control of several genes and results in specific neuronal and behavioral phenotypes. This finding suggests the importance of tight epigenetic regulation by cohesin at a specific time point in the developing brain. Thus, we have demonstrated a new epigenetic role for cohesion that affects development of neuronal networks.

Accumulating evidence suggests an association between dendritic abnormalities and higher-order brain function. Some genetic disorders, such as Down, Rett, and fragile X syndromes, are associated with dendrite and/or spine anomalies. In Down syndrome, dendrite branching and length are greater in infants compared with controls (Becker et al., 1986; Prinz et al., 1997), whereas branching, length, and spine density are all reduced in patients with Down syndrome (>30 yr old) compared with age-matched controls (Takashima et al., 1989; Becker et al., 1991). Dendritic arborization declines throughout life in individuals with Rett syndrome (Armstrong et al., 1995, 1998). Although neuronal density in the neocortex of individuals with fragile X syndrome is not different from normal controls, dendritic spines appear long and tortuous with prominent heads (Rudelli et al., 1985; Hinton et al., 1991). These variable synaptic complexity phenotypes might be consequences of altered formation, maturation, or pruning of synapses. In our experiment, greater dendritic arborization in the cortex of *Smc3^+/−^* mice might have resulted from deficient pruning of excess immature synapses or, alternatively, from compensating for fewer mature synapses. We cannot rule out the possibility that defective expression of factors that induce synapse formation could also have led to fewer mature synapses. To clarify these points, quantitative time course studies will be required.

We found that cohesin expression must be tightly controlled in the brain during development, and lower cohesin levels in the brain lead to global changes in gene expression at P7, which results in a specific neuronal and behavioral phenotype. Although the distribution change of cohesin in *Smc3^+/−^* mice was modest (Fig. 10), the occupancy of cohesin inside DEGs was decreased, and this may lead to the disruption of precise gene expression during the development of CNS. Interestingly, the expression of immune response–related genes, especially those related to the “response to interferons,” was altered in the brains of *Smc3^+/−^* mice (Fig. 8 D). Interferon-γ participates in the regulation of dendrite and synapse formation via STAT1 (Kim et al., 2002). We therefore postulate that cohesin supports dendrite and synapse formation at least partially through the regulation of the response of interferon-γ.

Furthermore, the disruption of epigenetic regulation leads to the up-regulation of immune genes and abnormal synaptic function (Feng et al., 2010). Deficiencies in the DNA methyltransferases DNMT1 and DNMT3 result in greater expression of immune genes, including STAT1, and lead to abnormal long-term plasticity (Feng et al., 2010). The up-regulation of STAT1 may be associated with synaptic plasticity (Tropea et al., 2006). We observed increased STAT1 expression in the cortex of *Smc3^+/−^* mice at P21 (Table S2, cluster 2). Additionally, several unregulated genes in *Smc3^+/−^* mice are shared by those in *Dnmt1/Dnmt3* double-knockout mice (Feng et al., 2010). Perturbations in DNA methylation may be relevant to intellectual disability in humans. Cells from patients with CdLS exhibit altered DNA methylation and cohesin binding (Liu et al., 2010). These observations suggest that cohesin regulates neuronal development in part through the regulation of immune gene expression. The disturbed synapse formation in *Smc3^+/−^* mice was partially, but not completely, rescued by STAT1 knockdown. These findings suggest that multiple genes contribute to these brain phenotypes and that cohesin strictly regulates the expression level of these genes.

In summary, decreased cohesin levels in mice led to defective dendrite and synapse formation in the brain and increased anxiety-like behavior. Many genes are transcriptionally controlled by cohesin, revealing a regulatory system of chromosomal architecture that is broadly responsible for neuronal network formation. Further studies will reveal specific temporal and spatial changes of chromosomal architecture during brain development.

## Materials and methods

### Mice

Mice were bred and maintained in the Institute of Experimental Animal Sciences at Osaka University Graduate School of Medicine. All experimental procedures were approved by the Institutional Committee of Osaka University.

A targeting vector in a pSP72 (Promega) vector backbone was constructed by first subcloning a 9.91-kb *Smc3*-specific genomic region from the 129/SvEv BAC clone RP23: 146L13. The region was designed such that the short homology arm extends 1.72 kb 3′ of exon 2, and the 5.75-kb-long homology arm ends 5′ of exon 1. A PGK-Frt/loxP-Neo cassette was inserted 177 bp downstream of exon 2, and a single *loxP* site was inserted 1.14 kb upstream of exon 1. The target region was 2.43 kb and included exons 1 and 2. The targeting vector was linearized with AscI and introduced into BA1 (C57BL/6 × 129/SvEv) hybrid ES cells by electroporation (inGenious Targeting Laboratory).

After selection with G418 antibiotic, the surviving clones were amplified by PCR and used for Southern blot analysis to identify recombinant ES clones. For Southern blot analysis, genomic DNA from the ES clones was digested with AvrII and BsrGI and hybridized with a probe targeted at either of the 5′ external regions, as indicated in Fig. 1. Five correctly targeted ES cell clones were identified among 200 neomycin-resistant colonies. Two mutated ES clones (#121 and #142) were microinjected into C57BL/6 blastocysts, and a colony of *Smc3^+^/flox* mice was successfully established from clone #121. Chimeric males (F0) were obtained and mated with C57BL/6J females to transmit the modified *Smc3* allele to the germline. To remove the Neo cassette, F1 heterozygotes were crossed with FLPe mice (B6.Cg-Tg(ACTFLPe)9205Dym/J, stock #003800; The Jackson Laboratory), generating offspring with a conditional *Smc3 loxP* allele (F2). The mice were backcrossed at least seven generations to the C57BL/6J strain (Charles River) for use in experiments.

Genotypes were determined by PCR of mouse tail DNA using NDEL1-F (5′-GGAAAGAATGTTGCCCTAGAAGT-3′) and NDEL2-R (5′-CGCTAGGCAAACATTCCACTAC-3′) for the wild-type (554 bp) and mutant (374 bp) alleles and LOX1-F (5′-GGTGGAATTTGGCCAGAGTTTCAG-3′), SDL2-R (5′-AATAGGTAGGTACTAGGGTCAGTCCC-3′), and AT2-R (5′-CTTAACTTACACCACTGGGCTCCA-3′) for the wild-type (434 bp) and mutant (373 bp) alleles.

To generate *Smc3^+/−^* mice, homozygous floxed mice (*Smc3 flox/flox*) were mated with B6.Cg-Tg(CAG-Cre)CZ-MO2Osb. (pCAGGS-Cre recombinase) transgenic mice (RBRC01828; Matsumura et al., 2004). Offspring carrying CAG-Cre and “floxed” (flanked by *loxP*) *Smc3* were backcrossed for at least seven generations with the wild-type C57BL/6 background. The viability of homozygous *Smc3*-knockout mice was examined by intercrossing *Smc3^+/−^* mice (Fig. 1 F). *Smc3^+/+^* mice were used as a control.

To generate neuron-specific *Smc3*-knockout mice, *Smc3 flox/flox* mice were crossed with telencephalin (TLCN)-Cre line D transgenic mice (Mitsui et al., 2007) to obtain offspring heterozygous for the floxed *Smc3* allele and hemizygous for the TLCN-Cre allele (TLCN-Cre; *Smc3^+^/flox*). To obtain homozygous floxed *Smc3*, TLCN-Cre; *Smc3^+^/flox* mice were crossed with *Smc3 flox/flox* mice. *Smc3 flox/flox* mice were used as a control.

Wnt1-Cre mice were obtained from The Jackson Laboratory (stock number 009107). tau-Cre mice were provided by A. Harada (Osaka University, Osaka, Japan; Muramatsu et al., 2008). Nestin-CreERT2 mice were provided by R. Kageyama (Kyoto University, Kyoto, Japan; Imayoshi et al., 2006). 4 mg tamoxifen was administered by oral gavage into pregnant mice at E10.5. The pups were fixed with 4% PFA at E18.5.

The Animal Research Committee of the Osaka University Graduate School of Medicine approved this study. All experiments were performed in accordance with the guidelines for animal experimentation from the Animal Research Committee of the Osaka University Graduate School of Medicine (permit number 24-067-005). All in vivo studies were performed under anesthesia.

### Behavioral analysis

Behavioral analyses were performed as previously described.

#### Animals and experimental design

Adult male mice at least 11 wk old were used for all behavioral analyses. Mice were housed under a 12-h light/dark cycle (lights on at 7:00 a.m.) with ad libitum access to food and water. Behavioral testing was performed between 9:00 a.m. and 5:00 p.m. After each test, the apparatus was cleaned with super-hypochlorous water to prevent bias caused by olfactory cues. The same mouse batch (group of mice) was used in all tests; all behavioral tests were separated from each other by at least 1 d. The raw data from the behavioral tests are not presented here but are available in the gene-brain-phenotyping database (http://www.mouse-phenotype.org/). All behavioral testing procedures have been approved by the National Institute for Physiological Sciences (Okazaki, Japan).

#### General health and neurological screen (Miyakawa et al., 2001)

The righting, whisker touch, and ear twitch reflexes were evaluated. Several physical features, including the presence of whiskers or bald patches in the coat, were also recorded.

#### Light/dark transition test (Takao and Miyakawa, 2006)

The apparatus used for the light/dark transition test was a cage (21 × 42 × 25 cm) divided into two sections of equal size by a partition with a door (O’Hara & Co.). One chamber was brightly illuminated (390 lux), whereas the other chamber was dark (2 lux). Mice were placed in the dark chamber and allowed to move freely between the two chambers with the door open for 10 min. The latency to first entry into the lit chamber, number of transitions, total distance traveled, and time spent in each chamber were recorded using ImageLD software (O’Hara & Co.; see Data analysis).

#### Open field test

Locomotor activity was measured using an open field test. Each mouse was placed in the corner of the open field apparatus (40 × 40 × 30 cm; Accuscan Instruments). The test chamber was illuminated at 100 lux. The total distance traveled, vertical activity (rearing measured by counting the number of photobeam interruptions), time spent in the center area (20 × 20 cm), and beam-break counts for stereotyped behavior were recorded. If an animal breaks the same beam (or set of beams) repeatedly, the monitor judges this behavior as stereotypy (as typically occurs during grooming and head bobbing, for example). The stereotypy count was the number of beam breaks that occurred during a period of stereotypic activity. Resting time was calculated as the difference between the total time and the time spent moving. The data were collected for 120 min.

#### Elevated plus maze test (Komada et al., 2008)

The elevated plus maze test consisted of two open arms (25 × 5 cm) and two enclosed arms of the same size with 15-cm-high transparent walls (O’Hara & Co.). The arms and central square were made of white plastic plates and were elevated 55 cm above the floor. To minimize the likelihood of animals falling from the apparatus, 3-mm-high Plexiglas walls surrounded the sides of the open arms. Arms of the same type were located opposite from each other. Each mouse was placed in the central square of the maze (5 × 5 cm), facing one of the closed arms. Mouse behavior was recorded during a 10-min test period. The number of entries into an arm and the time spent in the open and enclosed arms were recorded. The percentage of entries into open arms, time spent in open arms, number of total entries, and total distance traveled were analyzed. The data acquisition and analysis were performed automatically using ImageEP software (see Data analysis).

#### Rotarod test (Tsujimura et al., 2008)

Motor coordination and balance were tested using an accelerating rotarod (Ugo Basile North America). The test was performed by placing mice on rotating drums (3 cm in diameter) and measuring the time each animal was able to maintain its balance on the rod. The speed of the rotarod was accelerated from 4 rpm to 40 rpm over a 5-min period. The animals performed three trials per day on two consecutive days, and trials were separated by intervals of more than 1 h.

#### Hot plate test (Tsujimura et al., 2008)

The hot plate test was used to evaluate sensitivity to a painful stimulus. Mice were placed on a 55.0 ± 0.3°C hot plate (Columbus Instruments International), and the latency to the first hind paw response was recorded. The hind paw response was defined as either a foot shake or a paw lick.

#### Social interaction test in a novel environment

In the social interaction test, two mice of identical genotype that had previously been housed in different cages were placed in a box together (40 × 40 × 30 cm) and allowed to explore freely for 10 min (Tanda et al., 2009). Because a pair of mice was used as a sample in the test, the number of samples was half the usual number. Social behavior was monitored with a charge-coupled device camera connected to a Macintosh computer. Analysis was performed automatically using ImageSI software (see Data analysis). The total number of contacts, total duration of active contacts, total contact duration, mean duration per contact, and total distance traveled were recorded. If the two mice contacted each other and the distance traveled by either mouse was longer than 10 cm, the behavior was considered an “active contact.” Images were captured at three frames per second, and the distance traveled between two successive frames was calculated for each mouse.

#### Crawley’s sociability and preference for social novelty test

The test for sociability and preference for social novelty is a well-designed method (Crawley, 2004; Moy et al., 2004). The apparatus comprised a rectangular, three-chambered box with a lid containing an infrared video camera (O’Hara & Co.). Each chamber was 20 × 40 × 22 cm. The clear Plexiglas dividing walls had small openings (5 × 3 cm) to allow access from the middle chamber into each side chamber. In a corner of each side chamber was a small, circular wire cage (11-cm height, 9-cm diameter, 0.5-cm space between bars) that allowed nose contact between the bars but prevented fighting. A habituation session was not performed in the apparatus. An unfamiliar C57BL/6J male mouse (stranger 1) that had no prior contact with the subject mouse was placed in the cage in one of the side chambers of the cage. The placement of stranger 1 in the left or right side of the chamber was systematically alternated between trials. The subject mouse was then placed in the middle chamber and allowed to explore the entire social test box for 10 min. The amount of time spent in each chamber and within 5 cm of each wire cage was measured with the aid of a camera fitted on top of the box. After the first test, each mouse was tested in a second 10-min session to quantify social preference for a new stranger. A second, unfamiliar mouse (stranger 2) was placed in the cage of the chamber that had been empty during the first 10-min session. The test mouse thus had a choice between the first, already-investigated unfamiliar mouse (stranger 1) and the novel unfamiliar mouse (stranger 2). As in the first test, the amount of time spent in each chamber and within 5 cm of each wire cage during the 10-min session was recorded. All stranger mice were C57BL/6J male mice and were not littermates. Analysis was performed automatically using ImageCSI software (see Data analysis).

#### Prepulse inhibition/acoustic startle response (Tanda et al., 2009)

A startle reflex measurement system was used (O'Hara & Co.). A test session began by placing a mouse in a Plexiglas cylinder, where it was left undisturbed for 10 min. White noise was used as the startle stimulus, and the duration was 40 ms for all trial types. The startle response was recorded for 140 ms (measuring the response every 1 ms) starting with the onset of the prepulse stimulus. The level of background noise in each chamber was 70 dB. The peak startle amplitude recorded during the 140-ms sampling window was used as the dependent variable. A test session consisted of six trial types (i.e., two for startle stimulus-only trials, and four for prepulse inhibition trials). The intensity of the startle stimulus was 110 or 120 dB. The prepulse sound was presented 100 ms before the startle stimulus, and its intensity was 74 or 78 dB. Four combinations of prepulse and startle stimuli were used (74–110 dB, 78–110 dB, 74–120 dB, and 78–120 dB). Six blocks of the six trial types were presented in a pseudorandom order such that each trial type was presented once within a block. The mean intertrial interval was 15 s (range, 10–20 s).

#### Porsolt forced swim test (Takao et al., 2008)

To assess the depression-like behavior, we performed Porsolt forced test. The apparatus consisted of four Plexiglas cylinders (20 cm high × 10 cm diameter). An opaque panel separated the cylinders to prevent the mice from seeing each other (O’Hara & Co.). The cylinders were filled with water (23°C) to a height of 7.5 cm. A mouse was placed into the cylinder, and immobility and distance traveled were recorded over a 10-min test period. Images were captured at one frame per second. For each pair of successive frames, the area (pixels) within which the mouse moved was measured. When the area was below a certain threshold, the mouse was considered “immobile.” When the area equaled or exceeded this threshold, the mouse was considered “moving.” The optimal threshold was determined by adjusting it to the amount of immobility measured by human observation. Immobility lasting less than 2 s was not included in the analysis. Retention tests were administered 24 h after training. Data acquisition and analysis were performed automatically using ImagePS software (see Data analysis).

#### Barnes circular maze test (Imayoshi et al., 2008)

Barnes maze is a widely used behavioral test measuring spatial reference memory. The test was conducted on “dry land”—a white circular surface, 1.0 m in diameter, with 12 holes equally spaced around the perimeter (O’Hara & Co.). The circular open field was elevated 75 cm from the floor. A black Plexiglas escape box (17 × 13 × 7 cm) with paper cage bedding on the bottom was located under one of the holes. The hole above the escape box represented the target. The location of the target was consistent for a given mouse but was randomized across mice. The maze was rotated daily, with the spatial location of the target unchanged with respect to the distal visual room cues, to prevent a bias based on olfactory or proximal cues within the maze. Three trials per day were conducted for six successive days. On day 7, a probe trial was conducted without the escape box to confirm that this spatial task was acquired based on navigation using distal environment room cues. One trial was conducted immediately after the probe test, and another probe trial was conducted 1 wk later. The number of errors to reach the target hole and the time spent around each hole were recorded using ImageBM software (see Data analysis).

#### T-maze spontaneous alternation task (Ohira et al., 2013)

The spontaneous alternation task was conducted using a modified T-maze apparatus and an automated video-tracking system (O’Hara & Co.; Shoji et al., 2012). The apparatus was constructed of white plastic runways with 25-cm-high walls and was partitioned into six areas by sliding doors that could be automatically opened by sliding downward. The stem of the T was designated as area S2 (13 × 24 cm), and the arms of the T were designated as areas A1 and A2 (11.5 × 20.5 cm). Areas P1 and P2 were the connecting passageways from the arms (A1 or A2) to the start compartment (S1). Mice were placed in S1 and immediately subjected to a forced-choice run (pseudorandomly assigned to either the left or the right arm). Mice were held in one of the arms (A1 or A2) for 10 s. The door was then opened so that the mice could return to the start area (S1). They were held in S1 for 3 s and then subjected to a free-choice run in which they were given access to both arms. This sequence (trial) was repeated 10 times per day (cut-off time 7,200 s). The intertrial interval was 60 s. The percentage of trials in which mice entered the arm opposite the forced-choice run during the free-choice run was calculated as the percentage of correct responses. Data acquisition and data analysis were performed using ImageTM software (see Data Analysis).

#### Tail suspension test (Tsujimura et al., 2008)

The tail suspension test was performed over a 10-min test session according to cited procedures (Tsujimura et al., 2008). Mice were suspended 30 cm above the floor in a visually isolated area by adhesive tape that was placed ∼1 cm from the tip of the tail, and their behavior was recorded over a 10-min test period. Data acquisition and analysis were performed automatically using ImageTS software (see Data analysis).

#### Contextual and cued fear conditioning test (Tsujimura et al., 2008; Shoji et al., 2014)

On the training day (day 1), each mouse was placed in a test chamber (26 × 34 × 29 cm) inside a sound-attenuated room and allowed to explore freely for 2 min. A 60-dB white noise served as the conditioned stimulus and was presented for 30 s, followed by a mild (2 s, 0.3 mA) foot shock, which served as the unconditioned stimulus. Two more conditioned stimulus–unconditioned stimulus pairings were presented with a 2-min interstimulus interval. On day 2, context testing was conducted in the same chamber. Cued testing with altered context was conducted after conditioning using a triangular box (35 × 35 × 40 cm) made of white opaque Plexiglas, which was located in a different room. Data acquisition, control of stimuli (i.e., tones and shocks), and data analysis were performed automatically using ImageFZ software (see Data analysis). Images were captured at one frame per second. For each pair of successive frames, the area (pixels) in which the mouse moved was calculated. When this area was below a certain threshold (20 pixels), the behavior was considered “freezing.” When the area equaled or exceeded the threshold, the behavior was considered “nonfreezing.” The optimal threshold (number of pixels) was determined by adjusting to the amount of freezing measured by human observation. Freezing that lasted less than the defined threshold (2 s) was not included in the analysis.

#### Gait analysis (Yao et al., 2011)

The gait of adult mice during spontaneous walk/trot locomotion at velocities of 24 cm/s was analyzed using simultaneous video and reaction force analysis. Equivalent stride times for forepaws and hind paws were composed of a shorter stance and a longer swing time. Peak vertical reaction force increases with decreasing stance time, and that for the forepaws was ∼5% greater than that for the hind paws across the whole stance time range studied.

#### Marble-burying test

The marble-burying test was modified from a previous study (Njung’e and Handley, 1991). Mice were individually placed in transparent polycarbonate cages (12 × 27 × 9 cm) containing a 5-cm layer of fine bedding material (SLC; Hamamatsu Photonics) and 25 equally spaced glass marbles (1.5 cm in diameter). The number of marbles buried and the total distance traveled were recorded with a video camera for 30 min. When half the marble was in the fine bedding material, it was operationally defined as buried.

#### Novelty-induced hypophagia test

Anxiety-related behavior was also tested using the novelty-induced hypophagia test originally developed by Hen et al. (Dulawa and Hen, 2005; Meshi et al., 2006). Training consisted of daily sessions held for three consecutive days in which group-housed mice were exposed for 30 min daily to diluted (25%) sweetened condensed milk (Morinaga) from sippers attached to 5-ml pipettes in each cage (with food and drinking water available ad libitum). On day 4, mice were divided into two groups. In home-cage testing, mice were removed from their home cage and placed into a holding cage. After 30 min, one mouse was returned to the home cage fitted with a filled pipette under dim lighting (50 lux). The latency to drink and the volume consumed were recorded every 5 min for 30 min. For testing in the novel environment, mice were placed into new clean cages fitted with a filled pipette, white paper flooring (without shavings), and bright lighting (600 lux) provided by a 60-W light source located above the floor. The latency to drink and the volume consumed were recorded every 5 min for 30 min.

#### 24-h monitoring in the home cage

A system that automatically analyzes the locomotor activity of mice in the home cage was used (INNOVIVE). The system contains a home cage (29 × 18 × 12 cm) and a filter cage top, with an infrared video camera attached to the top of a 13-cm-high metal stand. Two mice of the same genotype that had been housed separately were placed together in a home cage. Their locomotor activity and social behavior were monitored for 1 wk. Output from the video camera was fed into a computer, and images were captured at a rate of one frame per second. Distance traveled was measured automatically using ImageHA software. Social interaction was measured by counting the number of particles detected in each frame: two particles indicated that the mice were not in contact with each other, and one particle (i.e., the tracking software could not distinguish two separate bodies) indicated contact between the mice. Using the same system, circadian rhythms of locomotor activity were also analyzed. Each mouse was individually housed for 1 wk under a 12-h light/dark cycle followed by 1 wk in constant darkness. Distance traveled was automatically measured and analyzed using ImageHA software (see Data analysis).

#### Data analysis

Behavioral data were obtained automatically by applications based on the public-domain ImageJ 1.46 program and modified for each test by Tsuyoshi Miyakawa (available through O’Hara & Co.). ImageLD, ImageEP, ImageFZ, ImageTM, and ImageHA are freely available at http://www.mouse-phenotype.org/software.html. Statistical analysis was performed using StatView (SAS Institute). The data were analyzed using a paired Student’s *t* test, one-way ANOVA, or two-way repeated-measures ANOVA. Values in graphs are expressed as the mean ± SEM.

### Golgi staining and Sholl analysis

Mice were maintained at a specific pathogen–free animal room in the Institute of Experimental Animal Sciences, Osaka University Graduate School of Medicine. Mice were housed three to five per cage in a room with a 12-h light/dark cycle with access to food and water ad libitum. Brains from P3w or P6w *Smc3^+/−^* mice, TLCN-Cre; *Smc3^+^/flox* mice, and gender-matched littermate-control mice were prepared using the standard Golgi-Cox impregnation technique using the FD Rapid GolgiStain kit (FD NeuroTechnologies, Inc.). Mice were anesthetized, and brain tissues were collected. 200-µm serial coronal sections were collected from control and *Smc3* mutant mice. In each hemisphere, pyramidal neurons with the soma in layer II/III were selected in the prefrontal cortex, which has been shown the relationship to higher-order brain function (bregma +2.10 to +0.70 mm; Paxinos and Franklin, 1997) and subsequently analyzed under higher magnification. Twelve cells were traced per mouse. Only fully impregnated pyramidal neurons that displayed dendritic trees without obvious truncation and that were isolated from neighboring impregnated neurons, were retained for analysis. For each selected neuron, the neuronal arbor was traced under an oil-immersion lens on a motorized microscope connected to a computer running Neurolucida Software (MBF Bioscience). A 40× lens was used to measure dendrites, and a 100× oil lens was used to observe spine morphology. The slides were examined by two masked observers. Sholl analysis was performed using NeuroExplorer (MBF Bioscience), and branch length, number of branches, neuronal complexity, spine length, and spine density were measured and analyzed. Two-way repeated-measures ANOVA was used for Sholl analysis. Statistical significance was set at P < 0.05. We used a minimal sample for this experiment in accordance with the National Centre for the Replacement, Refinement and Reduction of Animals in Research (London, UK).

### Histological analysis

Cryostat sections were incubated with blocking solution containing 5% bovine serum albumin and 0.1%–0.3% Triton X-100 in PBS for 1 h at room temperature and then incubated overnight with the appropriate antibodies at 4°C. Immunoreactivity was visualized using fluorescence-conjugated secondary antibodies. Sections were then mounted with coverslips and mounting medium. Antibodies to Smc3 (1:500; Abcam) and NeuN (1:2,000; EMD Millipore) were used. For Nissl staining, 20-µm-thick coronal brain sections prepared from *Smc3^+/+^* or *Smc3^+/−^* mice were stained with cresyl violet (MP Biomedicals). Skeletal samples prepared from P0.5 mice were stained with alizarin red and alcian blue using standard techniques.

### Microscope image acquisition

Images were acquired on a confocal microscope (FV1100) using FV10-ASW software (Olympus).

### Immunohistochemistry of autopsied brain tissue

The ethical committee of the Osaka Medical Center and Research Institute for Maternal and Child Health reviewed and approved the protocol for analysis of autopsied brain tissue. Brain tissue from stillborns with CdLS was obtained after death at 39 wk of pregnancy. Control brain tissue was obtained from stillborns free of neurological diseases after death at 40 wk of pregnancy. Tissues were fixed with 10% formalin and then embedded in paraffin. 6-µm-thick brain sections were cut, deparaffinized with xylene, and then rehydrated in ethanol. Sections were boiled in citric acid buffer and then subjected to immunofluorescence staining with antibodies to MAP-2 (1:500; Sigma-Aldrich), MBP (1:200; Abcam), Iba1 (1:200; Wako Pure Chemical Industries), and GFAP (1:200; Sigma-Aldrich). Sections were then washed with PBS and incubated with an Alexa Fluor 568–conjugated secondary antibody (1:1,000; Molecular Probes) at room temperature for 1 h.

### Electron microscopy

Mice were deeply anesthetized and transcardially perfused with PBS, pH 7.4, followed by ice-cold 2.5% glutaraldehyde in phosphate buffer, pH 7.4. The brains were removed, and the cortices were dissected and postfixed in glutaraldehyde solution at 4°C for 3 d. The samples were washed more than three times for 10 min each in 0.1 M phosphate buffer and then postfixed in 1% osmium tetroxide for 1 h at 4°C. Next, the samples were washed three times in distilled water for 10 min each. After en-bloc staining in 0.5% uranyl acetate in distilled water for 4 h to overnight, the samples were washed three times in distilled water for 10 min each. Samples were dehydrated using serial dilutions of ethanol (50%, 80%, 90%, 95%, and 100%) for 10 min each. Samples were then treated twice with propylene oxide for 20 min each and impregnated with 50:50 propylene oxide:epoxy resin overnight at room temperature. Finally, samples were impregnated with 100% epoxy resin, embedded in molds, and incubated for 48 h at 60°C. 1-µm semithin sections were then cut on an ultramicrotome (Reichert Ultracut E) and stained with 0.5% toluidine blue. From these, 80-nm thin striatal sections were cut on an ultramicrotome, mounted on 200-mesh copper grids, and poststained in 2% uranyl acetate in distilled water for 15 min and in Sato’s lead citrate stain for 7 min. Grids were examined by transmission electron microscopy (Hitachi H-7650). Images were acquired at 4,000× magnification using a Hamamatsu Photonics C4742-57-12ER CCD-based digital camera and Hitachi H-7650 control software, version 01.02. Postsynaptic densities were measured using ImageJ software (National Institutes of Health) by an observer who was blinded to the genotype of the samples.

### In situ hybridization

The cDNA fragments of mouse IFN-γ were amplified by RT-PCR using previously described in situ hybridization primers (Kimura et al., 2006). The amplified fragments were cloned into the pCR-blunt II-TOPO vector (Invitrogen). Serial sagittal sections, 16 µM thick were mounted on APS-coated glass slides (Matsunami Glass) and processed for in situ hybridization as previously described (Fujiwara et al., 2016).

### Western blotting

Mouse cortices were homogenized in lysis buffer containing 50 mM Tris-HCl, pH 7.5, 150 mM NaCl, 1 mM EDTA, 1.0% NP-40, and a protease inhibitor cocktail (Roche). Cell lysates were boiled in sample buffer for 5 min. The proteins were separated by SDS-PAGE and transferred onto polyvinylidene difluoride membranes (EMD Millipore). The membrane was blocked with 5% nonfat dry milk in PBS containing 0.05% Tween-20 (PBS-T) and then incubated with primary antibody diluted in PBS-T containing 1% nonfat dry milk for 1 h at room temperature or overnight at 4°C. After washing in PBS-T, the membrane was incubated with horseradish peroxidase–conjugated secondary antibodies. An enhanced chemiluminescence system (GE Healthcare) was used for detection. Signals were detected and quantified using an LAS-3000 image analyzer (Fuji Film). Class III β-tubulin was used as a loading control.

### RNA extraction, reverse transcription, and real-time PCR

Total RNA was extracted from astrocytes or the cortex with TRIzol (Invitrogen) and reverse transcribed using the High-Capacity cDNA Reverse Transcription kit (Applied Biosystems). mRNA expression was determined using a 7300 fast real-time PCR system (Applied Biosystems). TaqMan assays (Applied Biosystems) were used to quantitate *Smc1* (Mm 00484015_m1) and *Smc3* (Mm 00490624_m1) using the TaqMan Gene Expression Master Mix (Applied Biosystems). The SYBR Green assay was performed to quantify *Stat1* and *IFN-γ* expression. Primers for mouse Stat1 and IFN-γ were designed using Primer Express software (Applied Biosystems). The relative mRNA expression was calculated after normalization to the expression of glyceraldehyde 3-phosphate dehydrogenase (*GAPDH*) mRNA. Cycle threshold values (Ct values) were calculated by the ΔΔCt method to obtain fold differences.

### RNA extraction, cDNA library preparation, and RNA-sequencing

Total RNA was extracted from mouse cortex. The cDNA library was prepared using TruSeq RNA Sample Prep kit v2 (Illumina) for P1 samples, and TruSeq Stranded Total RNA with Ribo-Zero Gold kit (Illumina) for P3–P21 samples. All RNA-sequencing experiments were run on an Illumina HiSeq-2000, and RNA samples were sequenced with four replicates for P1 samples and three replicates for P3–P21 samples, according to the standard Illumina protocol, to create 31–65 × 10^6^ single-end reads.

### Read alignment and transcriptome processing

RNA-sequencing data analysis was performed with TopHat2 version 2.0.9 (Kim et al., 2013) and Cufflinks version 2.2.1 (Trapnell et al., 2013). RNA-sequencing reads for each library were independently mapped to the mouse reference sequence (NCBIM37/mm9) using TopHat2 with the options “-N 2 -G, Ensembl.gtf” (http://www.ensembl.org/info/data/ftp/index.html), which contains the annotations of both coding and noncoding genes. Further analysis to reveal differential expression in annotations and novel transcripts was performed with Cufflinks.

### GO analysis

Enrichment analysis of the GO terms for DEGs was performed using g:Profiler (Reimand et al., 2011). Among 382 DEGs, 290 genes with enough alignment for testing (test status was “OK”) were chosen for the clustering analysis after removing three sex-specific genes.

### ChIP-seq analysis

ChIP was performed as previously described (Izumi et al., 2015) except for homogenizing the fixed cells in 1% formaldehyde. LB1 (20 mM Tris-HCl, pH 7.5, 10 mM NaCl, 1 mM EDTA, 0.2% NP-40, 5 mM DTT, 1 mM PMSF). The cells were subjected to 10 strokes in a dounce homogenizer (Komata et al., 2014). Antibodies used for ChIP were as follows. Anti–histone H3 lysine-27 acetylation (H3K27Ac; Stasevich et al., 2014) and H3 lysine-4 trimethylation (H3K4me3) antibodies were provided by H. Kimura (Tokyo Institute of Technology, Tokyo, Japan). We also used antibody against Rad21, which has been described previously (Minamino et al., 2015). DNA from input and ChIP fractions were processed and sequenced using the Illumina HiSeq-2000 system, according to manufacturer’s instructions. In brief, DNA was sheared to an mean size of ∼150 bp by ultrasonication (Covaris), end-repaired, ligated to sequencing adapters, amplified, size-selected, and sequenced to generate single-end 66-bp reads. Sequenced reads were aligned to the mouse reference sequence (mm9) using Bowtie2 version 2.1.0 (Langmead and Salzberg, 2012) with default options. After filtering duplicated reads, peak calling and ChIP-seq data visualization were performed by DROMPA version 2.5.1 (Nakato et al., 2013). To calibrate ChIP-seq data for Rad21, we used the method described by Hu et al. (2015). The whole-cell extract from the mouse cortex was mixed with whole-cell extract from human RPE cells, and then ChIP was performed as described. Sequenced reads were aligned to mouse (mm9) and human (hg19) reference sequences. ChIP-seq profiles were multiplied by the occupancy ratio (OR) as previously described (Hu et al., 2015). The OR is calculated as follows: OR = (W_h_IP_m_)/(W_m_IP_h_), where W_m_ and IP_m_ are the number of mapped reads to only the mouse genome from input and IP fractions; W_h_ and IP_h_ are the number of mapped reads to only the human genome from input and IP fractions.

### Cell culture and siRNA

Mouse *Stat1* siRNA (stealth siRNA; Invitrogen) was used for knockdown experiments. Negative control siRNA (Ambion) with no sequence similarity to mouse genes was used as a control siRNA.

P1 mouse cortices were isolated and dissociated with 0.25% trypsin (Invitrogen) and 0.5 mg/ml DNase1 (Sigma-Aldrich) for 15 min at 37°C. DMEM (Invitrogen) containing 10% FBS was added, and the cells were centrifuged at 1,000 rpm for 4 min. Neurons were plated on poly-l-lysine–coated chamber slides and maintained in DMEM/F12 medium containing B27 supplement (Invitrogen) at 37°C in an atmosphere of 5% CO2. We obtained C57BL/6J mice (P1) from Japan SLC, Inc. The mice were bred and maintained in the Institute of Experimental Animal Sciences at Osaka University Graduate School of Medicine. The neurons were transfected with siRNA using a 4D-Nucleofector (Lonza). The cells were washed and resuspended in room temperature Nucleofector Solution. The cell–Nucleofector solution complex and siRNAs were then gently mixed and transferred into a cuvette, followed by nucleofection. Knockdown efficacy was analyzed after 72 h by real-time PCR. For immunocytochemistry, the cells were cultured for 21 d and fixed with 4% paraformaldehyde for 30 min. The cells were then permeabilized, and nonspecific sites were blocked by incubating with PBS containing 0.1% Triton X-100 and 5% BSA. The cells were incubated with anti-MAP2 (1:2,000; Sigma-Aldrich) and anti-PSD-95 (1:500; Thermo Fisher Scientific) antibodies diluted in a blocking solution overnight at 4°C. The cells were then washed in PBS and incubated with fluorescence-conjugated secondary antibodies for 1 h at room temperature. Because there was variability in PSD-95 punctate signals on neurons from the culture preparations, the number of PSD-95 signals from each group was normalized to the control group (*Smc3^+/+^* neurons transfected with control siRNA).

### Online supplemental material

Figs. S1 and S2 show the results of general behavioral tests and social behavior tests, respectively. Fig. S3 demonstrates the results of memory tests and locomotor activity in the home cage. Tables S1 and S2 are included as Excel files and show RNA-sequencing results and gene clustering results, respectively.

## Supplementary Material

Supplemental Materials (PDF)

Tables S1-S2 (zipped Excel files)
