# Supplementary material for: Decreased cohesin in the brain leads to defective synapse development and anxiety-related behavior
Source: J Exp Med. 2017 May 1;214(5):1431–52. doi: 10.1084/jem.20161517 (PMC5413336; doi:10.1084/jem.20161517)
Supplement: Supplemental Materials (PDF) [file JEM_20161517_sm.pdf]

The Journal of Experimental Medicine

SUPPLEMENTAL MATERIAL

Fujita et al., https://doi.org/10.1084/jem.20161517

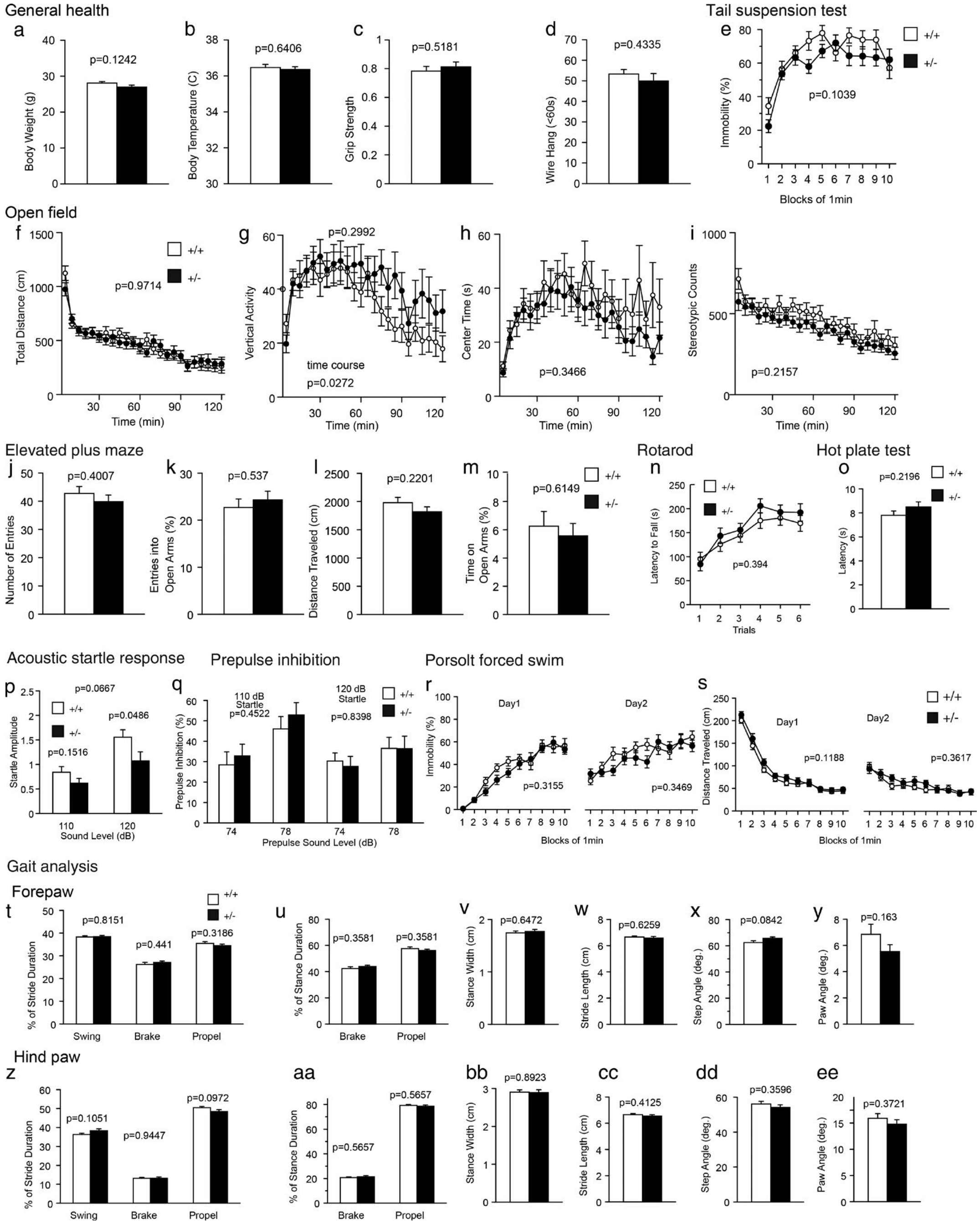

Figure S1. **General behavioral tests.** (a–d) General health. *Smc3*$^{+/+}$ mice and *Smc3*$^{+/-}$ mice had similar body weight (a), body temperature (b), and muscle strength (c and d). (e) Tail suspension test. No difference was found between *Smc3*$^{+/+}$ mice and *Smc3*$^{+/-}$ mice in the tail suspension test. *n* = 22–23 mice per genotype. (f–i) Open field test. *Smc3*$^{+/-}$ mice display similar total distance (f), vertical activity (g), center time (h), and stereotypic counts (i) compared with *Smc3*$^{+/+}$ mice. *n* = 23 mice per genotype. (j–m) Elevated plus maze test. The numbers of entries (j) entries into open arms (k), distance traveled (l), and time on open arms (m) of *Smc3*$^{+/-}$ mice were not significantly different from those of *Smc3*$^{+/+}$ mice. *n* = 23 mice per genotype. (n) Motor coordination and nociception tests. In the rotarod test, *Smc3*$^{+/+}$ mice and *Smc3*$^{+/-}$ mice showed similar levels of motor coordination. (o) In the hot plate test, sensory reaction was not significantly different between *Smc3*$^{+/+}$ and *Smc3*$^{+/-}$ mice. *n* = 23 mice per genotype. (p and q) Sensory-motor gating, hearing, and startle tests. No significant differences were found in the prepulse inhibition of the acoustic startle response between *Smc3*$^{+/+}$ and *Smc3*$^{+/-}$ mice. *n* = 23 mice per genotype. (r and s) Depression-like behavior test. In the Porsolt forced swim test, *Smc3*$^{+/-}$ mice displayed similar levels of immobility (r) and distance traveled (s) as *Smc3*$^{+/+}$ mice. *n* = 23 mice per genotype. (t–ee) Locomotion test. In the gait analysis, *Smc3*$^{+/-}$ mice showed similar percentages of stride in swing (t and z), percentages of stance duration (u and aa), stance width (v and bb), stride length (w and cc), step angle (x and dd), and paw angle (y and ee) as *Smc3*$^{+/+}$ mice. *n* = 22–23 mice per genotype. Values in graphs are expressed as the mean ± SEM.

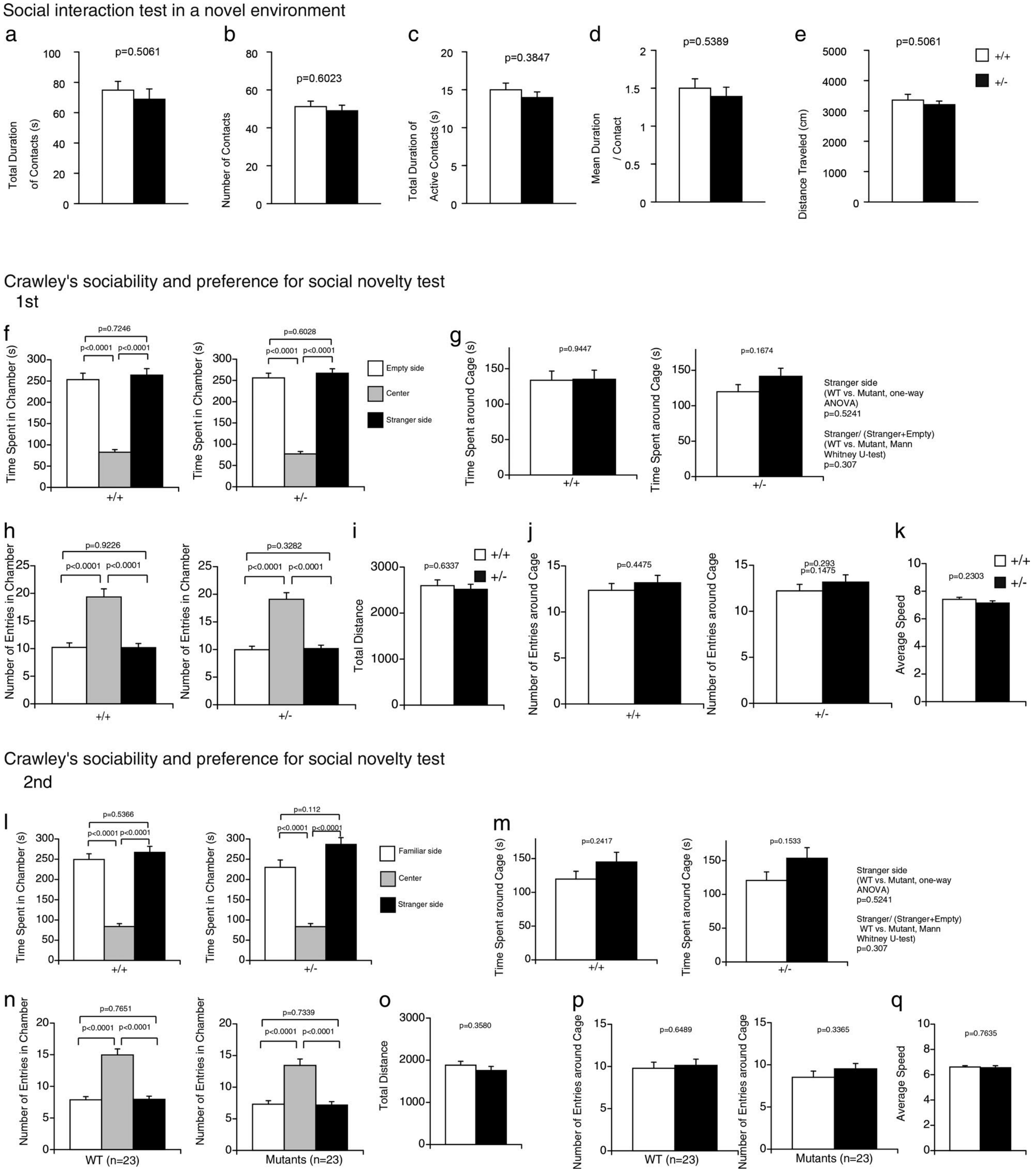

Figure S2. **Social behavior tests: social behavior test in a novel environment.** (a–e) In the social interaction test, no significant differences were found in total duration of contacts (a), number of contacts (b), total duration of active contacts (c), mean duration/contact (d), or distance traveled (e) between *Smc3*$^{+/+}$ and *Smc3*$^{+/-}$ mice. n = 11 mice per group. (f–q) Crawley's social behavior tests. In Crawley's sociability and preference for social novelty test, no significant differences were found between *Smc3*$^{+/+}$ and *Smc3*$^{+/-}$ mice in social interaction or social novelty-recognition behavior in either the first trial with one stranger mouse or in the subsequent trial with a second stranger mouse. n = 23 mice per genotype. Values in graphs are expressed as the mean ± SEM.

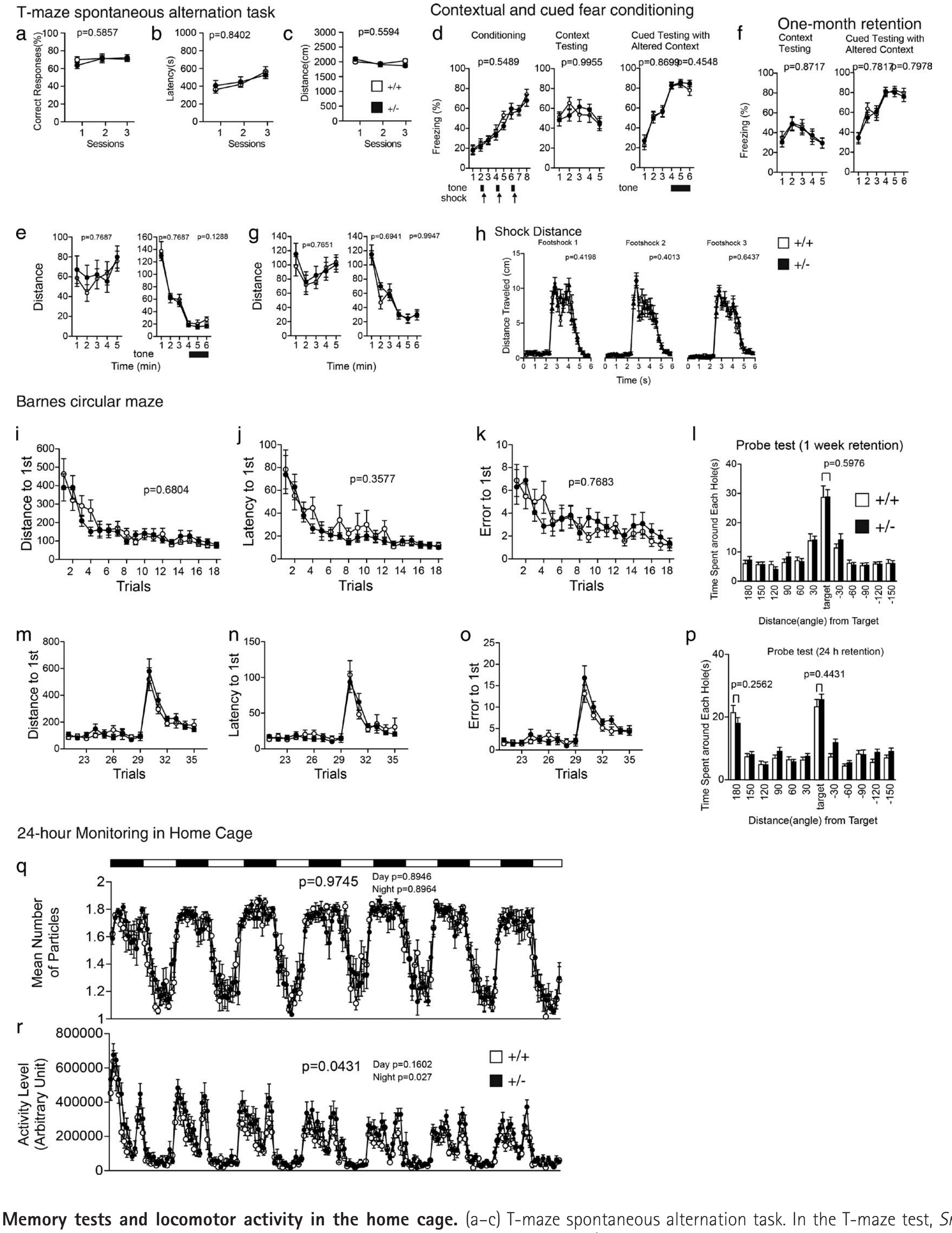

Figure S3. **Memory tests and locomotor activity in the home cage.** (a–c) T-maze spontaneous alternation task. In the T-maze test, *Smc3*$^{+/-}$ mice displayed percentages of correct responses (a), latency (b), and distance (c) similar to *Smc3*$^{+/+}$ mice. n = 22–23 mice per genotype. (d–h) Contextual and cued fear conditioning. Memory consolidation was examined by contextual and cued fear-conditioning tests. Memory retention was evaluated 1 mo after training. No significant differences were found between *Smc3*$^{+/+}$ and *Smc3*$^{+/-}$ mice in freezing (d and f), distance (e and g), or distance traveled after the shock (h). n = 22–23 mice per genotype. (i–k) Barnes circular maze test. In the Barnes circular maze test, *Smc3*$^{+/+}$ and *Smc3*$^{+/-}$ mice learned to locate the escape box during the training period. (l) The probe test was conducted 1 wk after the last training day. In the probe test, both *Smc3*$^{+/+}$ and *Smc3*$^{+/-}$ mice located the correct hole. (m–o) After the retention tests, mice received an additional 6 d of training on a memory reversal task in which the escape box location was reversed by 180°. (p) The probe test was conducted 24 h after the last reversal task-training day. n = 23 mice per genotype. (q and r) 24-h monitoring in home cage. In the 24-h home cage–monitoring test, *Smc3*$^{+/-}$ mice exhibited hyperactivity during the nighttime period. n = 9–10 mice per genotype. Values in graphs are expressed as the mean ± SEM.

Tables S1 and S2 are available as Excel files. Table S1 shows RNA-sequencing results. Table S2 shows gene clustering results.
